# A New Protocol for Ash Wood Modification: Synthesis of Hydrophobic and Antibacterial Brushes from the Wood Surface

**DOI:** 10.3390/molecules27030890

**Published:** 2022-01-28

**Authors:** Angelika Macior, Izabela Zaborniak, Paweł Chmielarz, Joanna Smenda, Karol Wolski, Ewa Ciszkowicz, Katarzyna Lecka-Szlachta

**Affiliations:** 1 Doctoral School of Engineering and Technical Sciences, Rzeszow University of Technology, Al. Powstańców Warszawy 8, 35-959 Rzeszów, Poland; d519@stud.prz.edu.pl; 2 Department of Physical Chemistry, Faculty of Chemistry, Rzeszow University of Technology, Al. Powstańców Warszawy 6, 35-959 Rzeszów, Poland; i.zaborniak@prz.edu.pl; 3 Faculty of Chemistry, Jagiellonian University, Gronostajowa 2, 30-387 Kraków, Poland; joanna.smenda@doctoral.uj.edu.pl (J.S.); wolski@chemia.uj.edu.pl (K.W.); 4 Department of Biotechnology and Bioinformatics, Faculty of Chemistry, Rzeszow University of Technology, Al. Powstańców Warszawy 6, 35-959 Rzeszów, Poland; eciszkow@prz.edu.pl (E.C.); szlachta@prz.edu.pl (K.L.-S.)

**Keywords:** ash wood, methacrylates, hydrophobicity, antibacterial surfaces, ARGET SI-ATRP

## Abstract

The article presents the modification of ash wood via surface initiated activators regenerated by electron transfer atom transfer radical polymerization mediated by elemental silver (Ag^0^ SI-ARGET ATRP) at a diminished catalyst concentration. Ash wood is functionalized with poly(methyl methacrylate) (PMMA) and poly(2-(dimethylamino)ethyl methacrylate) (PDMAEMA) to yield wood grafted with PMMA-*b*-PDMAEMA-Br copolymers with hydrophobic and antibacterial properties. Fourier transform infrared (FT-IR) spectroscopy confirmed the covalent incorporation of functional ATRP initiation sites and polymer chains into the wood structure. The polymerization kinetics was followed by the analysis of the polymer grown in solution from the sacrificial initiator by proton nuclear magnetic resonance (^1^H NMR) and gel permeation chromatography (GPC). The polymer layer covalently attached to the wood surface was observed by scanning electron microscopy (SEM). The hydrophobic properties of hybrid materials were confirmed by water contact angle measurements. Water and sodium chloride salt aqueous solution uptake tests confirmed a significant improvement in resistance to the absorption of wood samples after modification with polymers. Antibacterial tests revealed that wood-QPDMAEMA-Br, as well as wood-PMMA-*b*-QPDMAEMA-Br, exhibited higher antibacterial activity against Gram-positive bacteria (*Staphylococcus aureus*) in comparison with Gram-negative bacteria (*Escherichia coli*). The paper presents an economic concept with ecological aspects of improving wood properties, which gives great opportunities to use the proposed approach in the production of functional hybrid materials for industry and high quality sports equipment, and in furniture production.

## 1. Introduction

Wood is a material with an uneven structure, and its quality properties depend on considering the anatomical direction. Therefore, wood is defined as a heterogeneous, multidirectional, i.e., anisotropic, material [1,2,3]. The structure of the wood varies depending on the type. The main division is softwood and hardwood, but shrinkage, sapwood, and heartwood, mature and juvenile are also distinguished [1,2,4]. The surface quality of such material plays an important role in their finishing and bonding properties.

Ash (*Fraxinus excelsior*) is a very popular species of hardwood. As a material, it shows high flexibility, resistance to shock and cracking, high lightness, and shock absorption, which is favorable for many applications in the furniture industry, e.g., chairs, armchairs and products bent, paneling, flooring, interior joinery, veneers for furniture and wardrobes and the production of sports articles, handles, baseball bats and tennis rackets [5,6]. However, despite its functional properties, all types of wood, including ash wood, depending on their end use, are subject to complex processes of physical, chemical, and mechanical degradation. The exposed wood surface becomes rough as the grain surfaces degrade and cracks appear on the wood surface, making the material unsuitable for long term use [5,7,8]. According to certain quality characteristics of the wood, appropriate technologies based on physical or chemical treatments are needed to correct its defects [7,8,9].

Chemical modifications involve the use of harmful chemicals (phenol-formaldehyde, melamine-formaldehyde resins, alkyd paints—a source of volatile organic compounds harmful to the environment, VOCs) or solvents that can cause environmental and health problems when processing and using modified wood [8,10,11]. One of the innovative techniques used to improve the mechanical properties of wood quality, i.e., modulus of rupture, abrasion, compressive strength, and hardness, is wood modification by the in situ polymerization of various monomers [7,8,12]. This approach allows the implementation of innovative solutions in functional wood based materials, and the resulting products are generally known as wood–polymer composites. The versatility and potential of this approach show that the native wood structure is preserved despite being modified with different types of polymer chains [8,13]. Both raw wood and wood fibers (elements that are extracted from wood) can be modified to improve selected performance criteria, e.g., hydrophobicity [14,15,16,17,18], strength [19,20,21,22,23], as well as antimicrobial properties [24,25,26,27,28,29].

Due to the large variety of functional groups (mainly hydroxyl and phenolic groups) in its structure, wood is an excellent candidate for chemical modification by controlled radical polymerization, i.e., atom transfer radical methods (ATRP) [30,31,32]. Compared to the aforementioned treatment of wood with monomers solutions, these techniques covalently bind polymers to the wood structure. This enables the incorporation of high molecular compounds (polymers) into the wood structure, which in turn leads to a significant improvement in its properties. In addition, it is possible to incorporate polymers with different characteristics by polymerizing different types of monomers simultaneously, giving the wood more than one property. Moreover, ATRP methods enable the creation of polymer chains with a strictly designed structure and architecture [33,34]. Therefore, polymers and biopolymers produced by the ATRP technique are characterized by a narrow molecular weight distribution (*M*_w_/*M*_n_), control over their molecular weight (MW) [30,35], and topology (geometry) [36,37] to obtain structures ranging from linear chains, stars, cyclic structures, combs, and regular polymer networks [32,37,38,39].

Up to now, ATRP methods have been successfully used for raw wood modification only a few times [16,18,40,41,42,43,44]. Poplar wood [16,40], spruce wood [41,44], waterlogged archaeological wood [42,43] and fir wood [18] were grafted with polymers to improve their properties, i.e., hydrophobicity [16,18,43], resistance to attack by fungi and termites, and antibacterial characteristics [40], controlled swelling behavior and tunable wettability [41,44], dimensional stability of wood, therefore preventing shrinkage, cracking and deformation [42]. The approaches ranged from both the classical high catalyst concentration ATRP technique [41,44] to the low ppm ATRP concepts, mainly activators regenerated by electron transfer (ARGET) ATRP with ascorbic acid as a reducing agent [16,40,42,43]. Our previous investigation was focused on the modification of fir wood using supplemental activator and reducing agent (SARA) ATRP with copper wire (Cu^0^) as a reducing agent, polymerizing hydrophobic monomers from wood structure [18]. Among the aforementioned techniques, SARA ATRP uses a reducing agent in the form of a solid chemical compound, thus, it is easily removed from the reaction mixture after polymerization and does not penetrate the wood structure. In the SARA ATRP technique, the balance between the active and inactivated forms of the catalytic complex is established using metallic copper, which can adopt three oxidation states and acts as an auxiliary activator, which is described by the mechanism of comproportionation and disproportionation. This reaction system caused side reactions [18], and the control over the methyl methacrylate (MMA) polymerization was low. In this consideration, ARGET ATRP with the use of silver as a reducing agent turned out to be useful for the synthesis of functional macromolecules with a controlled and complex architecture [45,46]. Unlike metals used in the SARA ATRP technique, Ag^0^ takes two oxidation states, therefore it is only an electron donor and does not participate in auxiliary activation, eliminating a number of side reactions [47,48].

The presented paper is focused on the grafting of methacrylic polymers with hydrophobic characteristics and precursors for antibacterial properties from ash wood by surface initiated ARGET ATRP (SI-ARGET ATRP) techniques with Ag^0^ as a reducing agent. To transform a hydrophilic wood surface into a hydrophobic surface, this study focused on the grafting of poly(methyl methacrylate) (PMMA) onto the wood surface, while poly(2-(dimethylamino)ethyl methacrylate) (PDMAEMA) was used as a precursor to create antibacterial surfaces. Elemental silver can be reactivated with facile treatments and reuse, thus, it constitutes an economic and industrial relevant approach. In the paper, the properties of 2-(dimethylamino)ethyl methacrylate (DMAEMA) as an instinct reducing agent was also presented [49]. DMAEMA was polymerized from the initiation sites on the wood without the addition of any additional reducing agent. The reaction proceeds by radical polymerization, then the tertiary amine groups are quaternized by an alkyl halide—bromoethane, and the quaternary ammonium groups on the wood surface have proven to be antibacterial. Currently, the interest in surfaces with antibacterial properties has increased significantly. Considering human health and life, it is important to keep materials sterile. Such materials inhibit the growth of bacteria, viruses, and fungi, and at the same time allow the destruction of microorganisms. Therefore, in recent years, there has been a significant increase in interest in surfaces with antibacterial properties in hospitals and pharmacies, and in the food and cosmetics industries [49,50].

## 2. Results and Discussion

### 2.1. The Concept of a “Grafting from” Approach for the Modification of Ash Wood Cubes

**Scheme 1:**
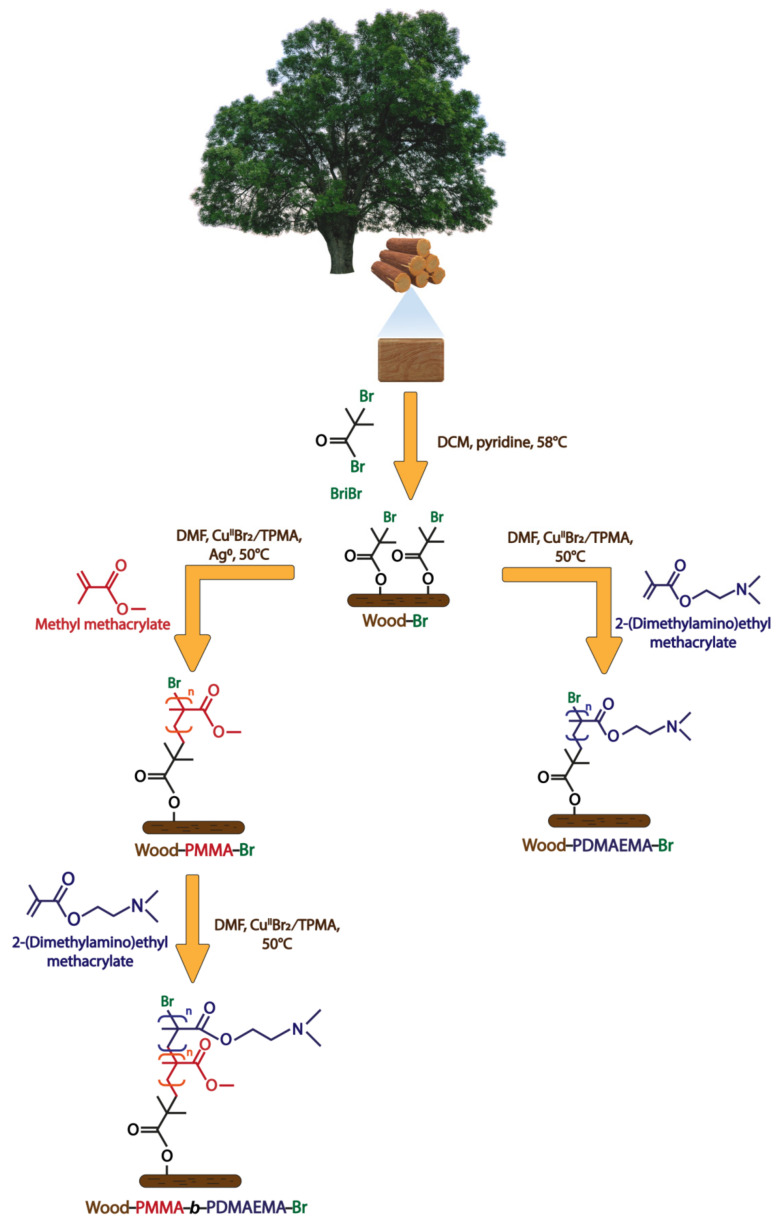
A two-step synthetic route for the preparation of a wood–polymer composite. Abbreviations: Ag^0^—sliver wire, BriBBr—2-bromoisobutyryl bromide, Cu^II^Br_2_/TPMA—catalytic complex, DCM—dichloromethane, TPMA—tris(2-pyridylmethyl)amine), wood-Br—brominated wood, wood-PMMA-Br—wood grafted with PMMA, wood-PMMA-*b*-PDMAEMA-Br—wood grafted with block copolymer composed of PMMA and PDMAEMA.

Herein, the dual properties (hydrophobic and antibacterial) of functional polymer-wood composites are incorporated into ash wood using the controlled atom transfer radical polymerization of MMA and DMAEMA from brominated wood cubes. The modification of ash wood based on a two-step synthesis was performed according to the “grafting from” concept. Hydroxyl groups located in the wood cell wall, i.e., cellulose, hemicellulose, and lignin, were esterified with α-bromoisobutyryl bromide creating ATRP initiation sites, followed by the polymerization of MMA or DMAEMA via SI-ATRP from brominated wood cubes to prepare hydrophobic and antibacterial polymer grafts in the form of homopolymers or block copolymers (Scheme 1).

The polymerizations of MMA were carried out by the SI-ARGET ATRP approach using silver wire (Ag^0^) as a reducing agent (Table 1), while the DMAEMA serves as an internal reducing agent, therefore, the syntheses were performed by ARGET ATRP with Ag^0^, but also without an additional chemical reducing agent (Table 2).

### 2.2. Preparation of Wood–Polymer Composites with Dual Characteristics

The SI-ARGET ATRP approach using silver wire with varying catalyst concentrations was used to synthesize PMMA chains grafted to the wood structure (Table 1). This process is based on the transfer of an electron from silver, establishing a dynamic equilibrium between the two forms of the catalyst to ensure the controlled manner of polymerization processes [46,48]. A sacrificial initiator—ethyl 2-bromoisobutyrate (EBiB), was added to the reaction mixture to track the kinetics of the polymerization. Thus, the polymerization occurred both from the initiator attached to the wood surface and from the sacrificial initiator. The molecular weight and molecular weight distribution of the linear homopolymers were investigated by sampling the solution mixture during the polymerization, assuming that the polymers were growing at the same constant rate in the reaction mixture as from the surface of the wood. For the synthesis of polymethacrylates in a controlled manner, other types of initiators are usually used, e.g., 2-bromopropionitrile (BPN) [51], however, the wood samples were modified with 2-bromoisobutyryl bromide (BriBBr), mimicking the structure of EBiB, which was used for the polymerization in solution.

**Figure 1:**
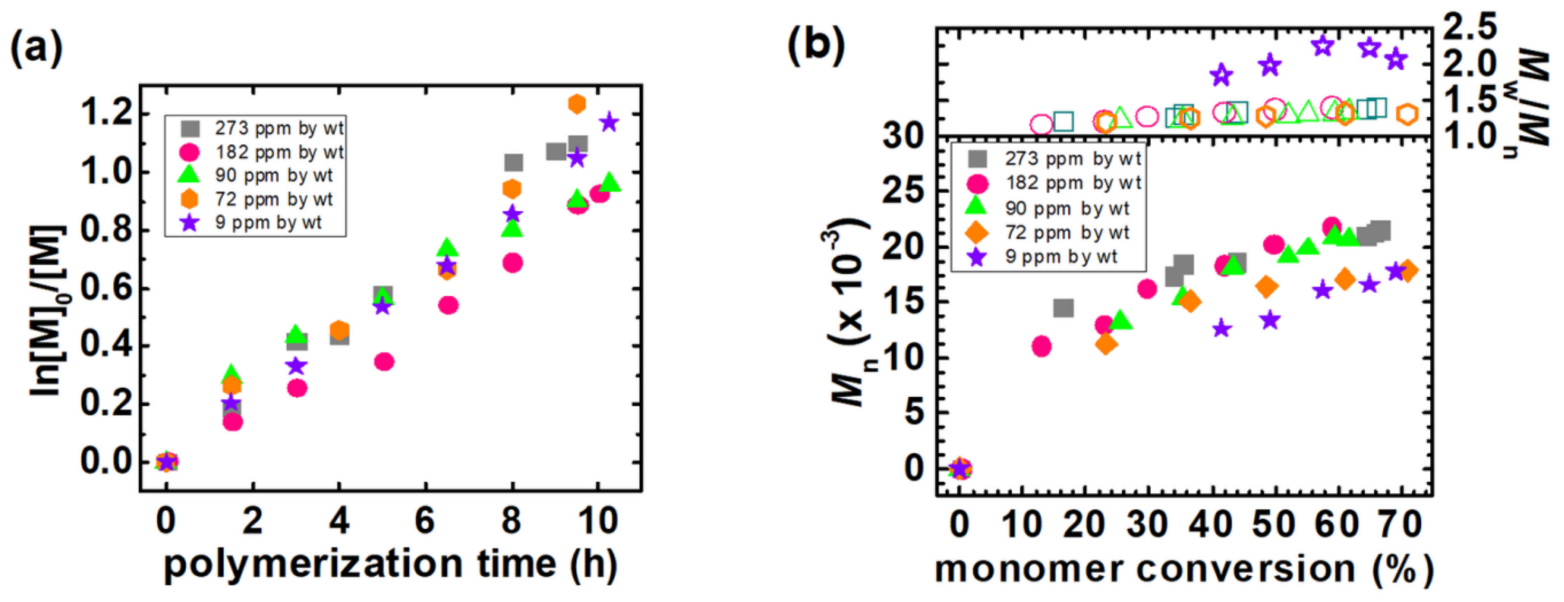
Polymerization of MMA varying concentrations of the catalytic complex: (**a**) first order kinetics plot of monomer conversion vs. polymerization time, and (**b**) *M*_n_ and *M*_w_/*M*_n_ vs. monomer conversion (Table 1).

**Figure 2:**
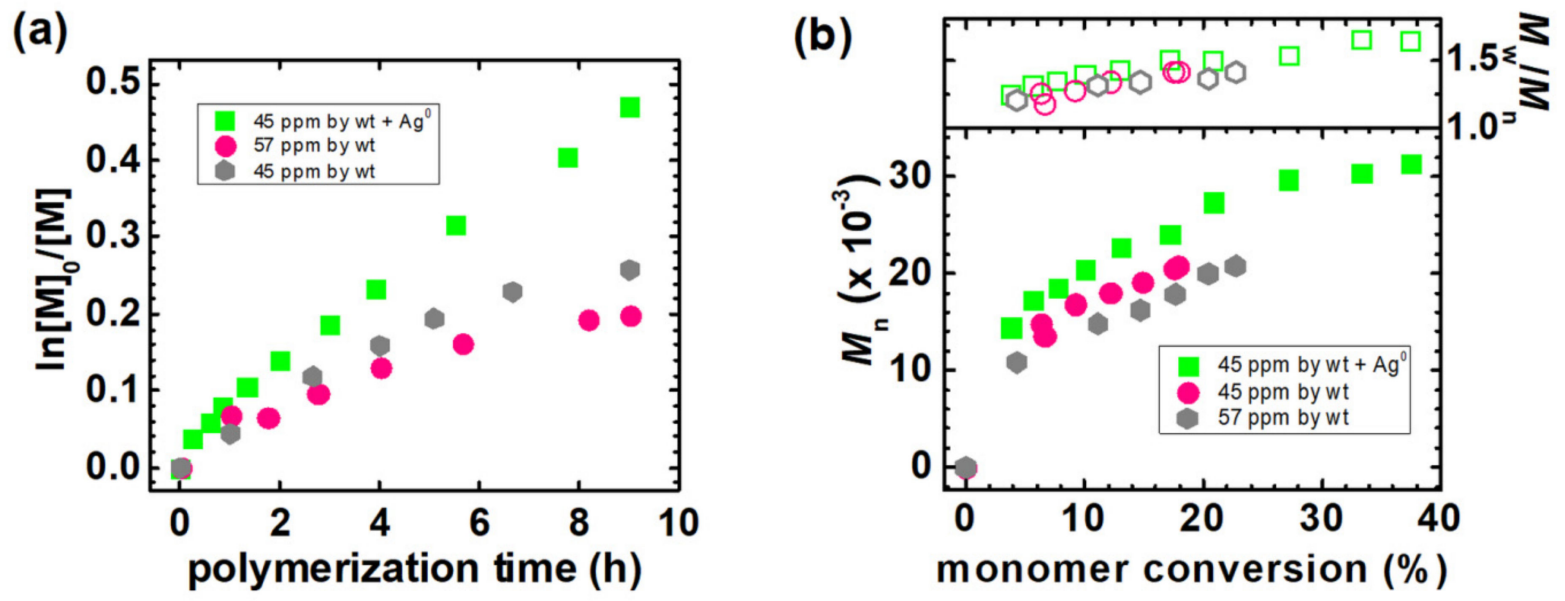
Polymerization of DMAEMA by SI-ARGET ATRP concept with silver wire as a reducing agent and applying self-reducing properties of the monomer: (**a**) first order kinetics plot of monomer conversion vs. polymerization time, and (**b**) *M*_n_ and *M*_w_/*M*_n_ vs. monomer conversion (Table 2).

The polymerization of MMA from brominated wood was described by the linear dependence presented in the first order kinetics plot (Figure 1a). While the molecular weight vs. monomer conversion graphs (Figure 2b) are characterized by a linear course up to approx. 40%, then, a slight deviation from the linear growth towards lower molecular weights was observed. At the high catalyst concentration, only a slight change in the molecular weight of the polymer was observed after 35% conversion, despite the steadily increasing MMA conversion. This is related to the termination processes, which in the case of PMMA is most often associated with the disproportionation reaction due to steric hindrance or chain activity transfer [52,53] (Figure 1b). While too low a concentration of the catalyst complex (9 ppm by wt) does not ensure a controlled course of the polymerization. The polymer molecular weight changed slightly after reaching 40% conversion. Insufficient catalyst loading may cause chain activity transfer to, e.g., monomer or solvent, consequently leading to the termination of the grown polymers. Uncontrolled polymerization provided high dispersity final products (*M*_w_/*M*_n_ > 2, Table 1, entry 5). Controlled polymerization was achieved using 72 ppm by wt of catalyst, as indicated by a narrow molecular weight distribution of the final polymer product (*M*_w_/*M*_n_ = 1.31, Table 1, entry 4). Based on GPC analysis, it was found that the theoretical number average molecular weights (*M*_n,theo_) and the molecular weights determined by chromatographic analysis (*M*_n,app_) for the obtained methacrylates, were different. This may be due to the consumption of the monomer by the surface of the wood, which has a porous structure, and the initiator is not only on the surface of the wood but also in the interior (see details in Supplementary Materials: Section S1). Although there is more EBiB initiator in the reaction medium than attached to one wood cube (in the range of 2.9–4.7 times more), there are always several wood samples in the mixture from which polymerization takes place.

To impart antibacterial properties to the wood, DMAEMA was polymerized from wooden cubes grafted with PMMA to create a second block as a precursor with antibacterial properties. Initially, a set of optimization reactions were performed by the polymerization of DMAEMA from brominated wood, forming a homopolymer coating on the wood surface (Table 2).

Similar to MMA polymerization, SI-ARGET ATRP with a silver wire was used to add a PDMAEMA block. The polymerization was characterized by a linear dependence of ln([M]_0_/[M]) on time (Figure 2a), reaching approx. 37% of DMAEMA conversion. As shown in the MW vs. conversion plot, at the initial step of the synthesis the *M*_n_ rapidly grew, followed by a deviation from the linearity towards lower MWs, reaching almost a plateau at approx. 30% of monomer conversion–the molecular weight of the polymer only slightly increased despite the monomer being consumed (Figure 2b and Figure S2a). The polymerization provided the final product with high dispersity (*M*_w_/*M*_n_ = 1.64, Table 2, entry 1). Since the DMAEMA monomer serves as a reducing agent due to the presence of the tertiary amine group [49], the polymerization was carried out without any additional chemicals to reduce a catalyst. The syntheses with 45 ppm by wt of a copper based catalyst (Table 2, entry 2) without silver wire were characterized by first order kinetics, however, approx. 2-fold lower polymerization rate was observed. Nevertheless, the lack of elemental silver in the reaction mixture resulted in a final product with a narrower molecular weight distribution (*M*_w_/*M*_n_ = 1.42, Table 2, entry 2, Figure S2b), therefore, a more controlled structure of the polymer. Increasing the catalyst concentration to 57 ppm by wt (Table 2, entry 3) resulted in a slightly higher polymerization rate, obtaining a monomer conversion of 23%, however, it had a negligible impact on the quality of the final product, a polymer with a comparable dispersity was obtained (*M*_w_/*M*_n_ = 1.41, Table 2, entry 3, Figure S2c).

Hence, the procedure with 45 ppm by wt of catalyst loading was used to synthesize the second PDMAEMA block from hydrophobic PMMA polymer grafts attached to the wood surface (Table 3, entry 2). The first PMMA block of the polymer–wood composite was synthesized according to the optimized procedure presented in Table 1, entry 4.

**Figure 3:**
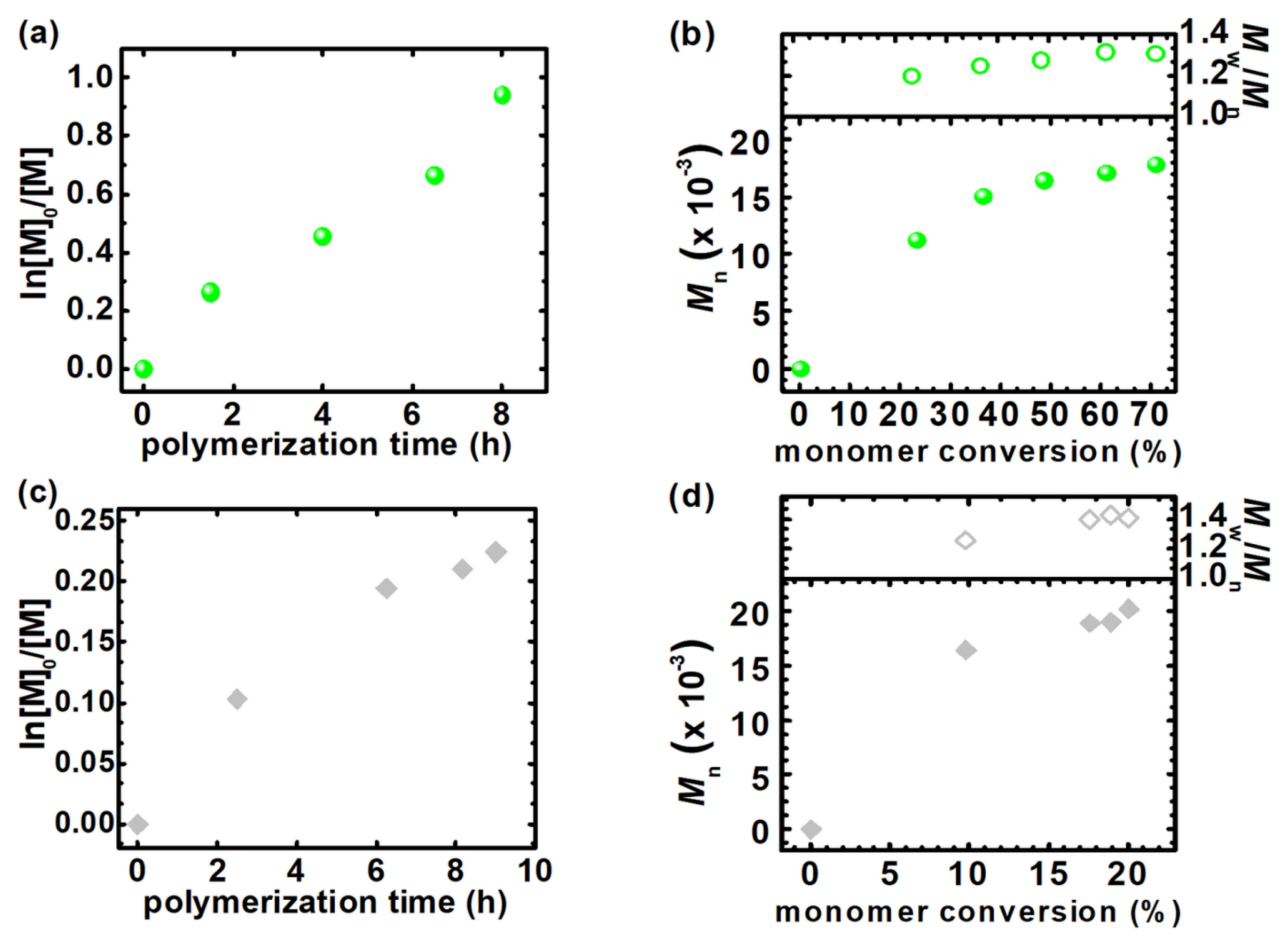
First order kinetics plot for (**a**) MMA and (**c**) DMAEMA polymerization; *M*_n_ and *M*_w_/*M*_n_ vs. monomer conversion plot for (**b**) MMA and (**d**) DMAEMA polymerization.

Linear plots of first order kinetics as a function of polymerization time (Figure 3a,c) and polymer growth with monomer conversion (Figure 3b,d) were observed. The results of the kinetic studies show the controlled polymerization of MMA and DMAEMA, resulting in a polymer product formed in solution with a low dispersity at each stage of polymerization (*M*_w_/*M*_n_ = 1.31 and 1.41 for PMMA and PDMAEMA, respectively).

The structures of PMMA and PDMAEMA linear polymers growing in the reaction mixture were confirmed by proton nuclear magnetic resonance (^1^H NMR) analysis (Figures S4 and S5).

**Figure 4:**
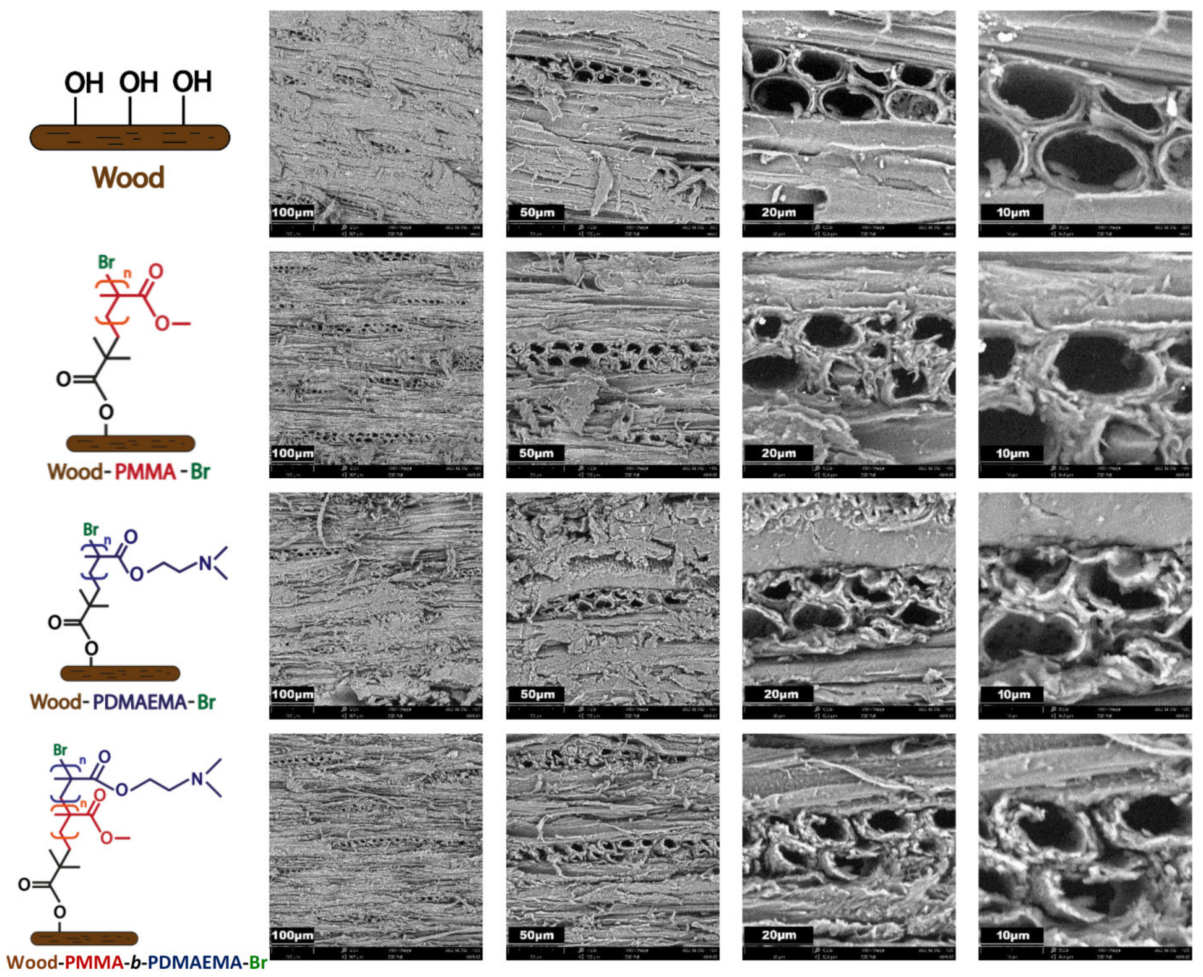
SEM images composed of unmodified wood, modified ash wood fibers wood-PMMA-Br (72 ppm by wt), wood-PDMAEMA-Br (45 ppm by wt), and wood-PMMA-*b*-PDMAEMA-Br captured with different magnifications (530×, 1400×, 4300×, 7800×).

**Figure 5:**
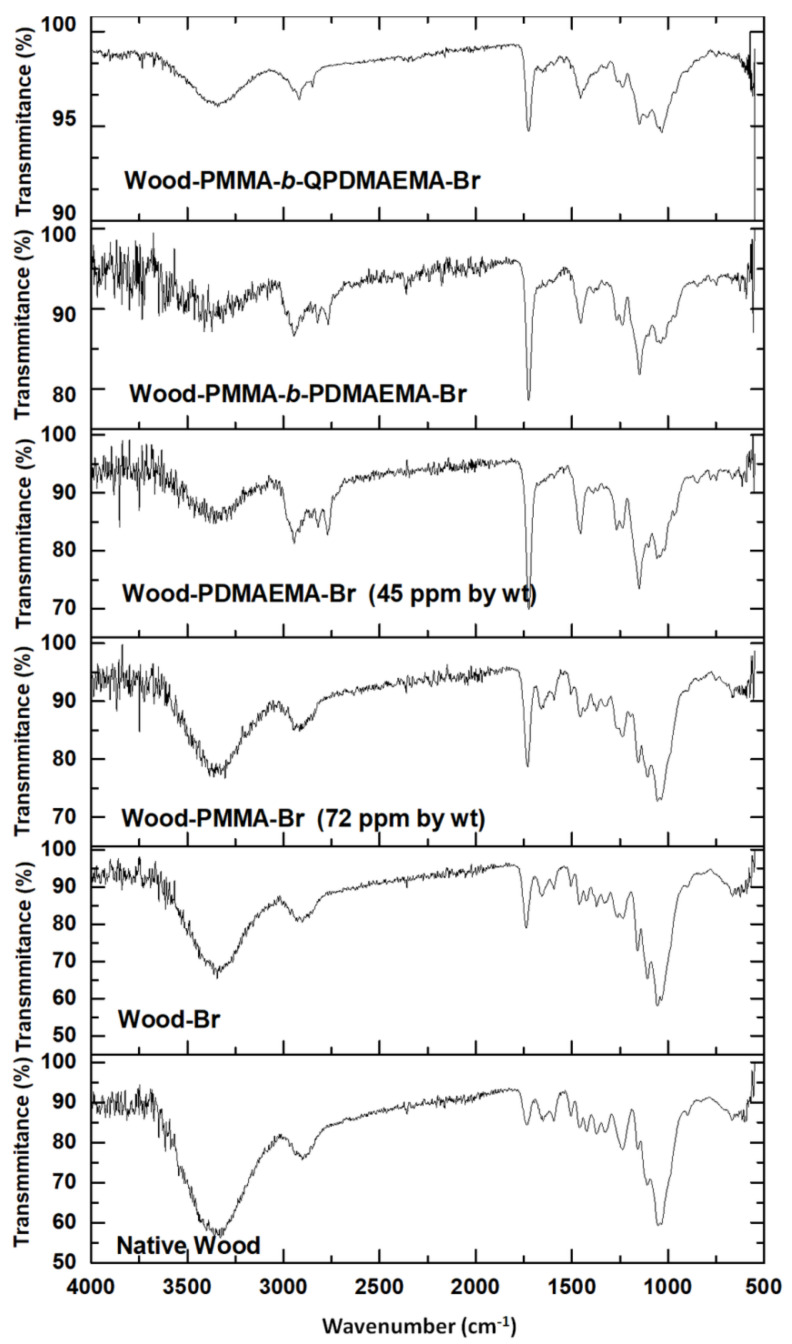
FT-IR spectra of native wood (untreated), wood esterified with ATRP initiator (wood-Br), and grafted with PMMA (wood-PMMA-Br), followed by polymerization the second PDMAEMA block (wood-PMMA-*b*-PDMAEMA-Br) and quaternization process (wood-PMMA-*b*-QPDMAEMA-Br).

The morphology of the wood fibers, both before and after polymer modification, was analyzed by scanning electron microscopy (SEM) (Figure 4 and see details in Supplementary Materials: Section S5) and the atomic force microscopy (AFM) analysis of the brushes grafted from silicon wafers (see details in Supplementary Materials: Section S6). Furthermore, the Fourier transform infrared (FT-IR) spectroscopy method was used to obtain complementary information about the modification of the wood surface (Figure 5 and see details in Supplementary Materials: Section S4).

The SEM image shows a continuous PMMA layer on top of the wood and similar results were observed for PDMAEMA brushes grafted from brominated wood (Figure 4). A clear difference is observed between the surface morphology of unmodified wood and those of the modified polymers. The tests of two different fiber surfaces, both before and after modification, clearly show the presence of polymer structures on the surface of the modified ash wood. Abundant polymer matter was found on the vessel walls. It is visible especially on the high magnification SEM micrographs of modified wood. The grafting is not spatially homogeneous due to the heterogeneity of the grafted initiator on the wood surface.

### 2.3. The Unique Properties of Wood Grafted with Polymers

**Figure 6:**
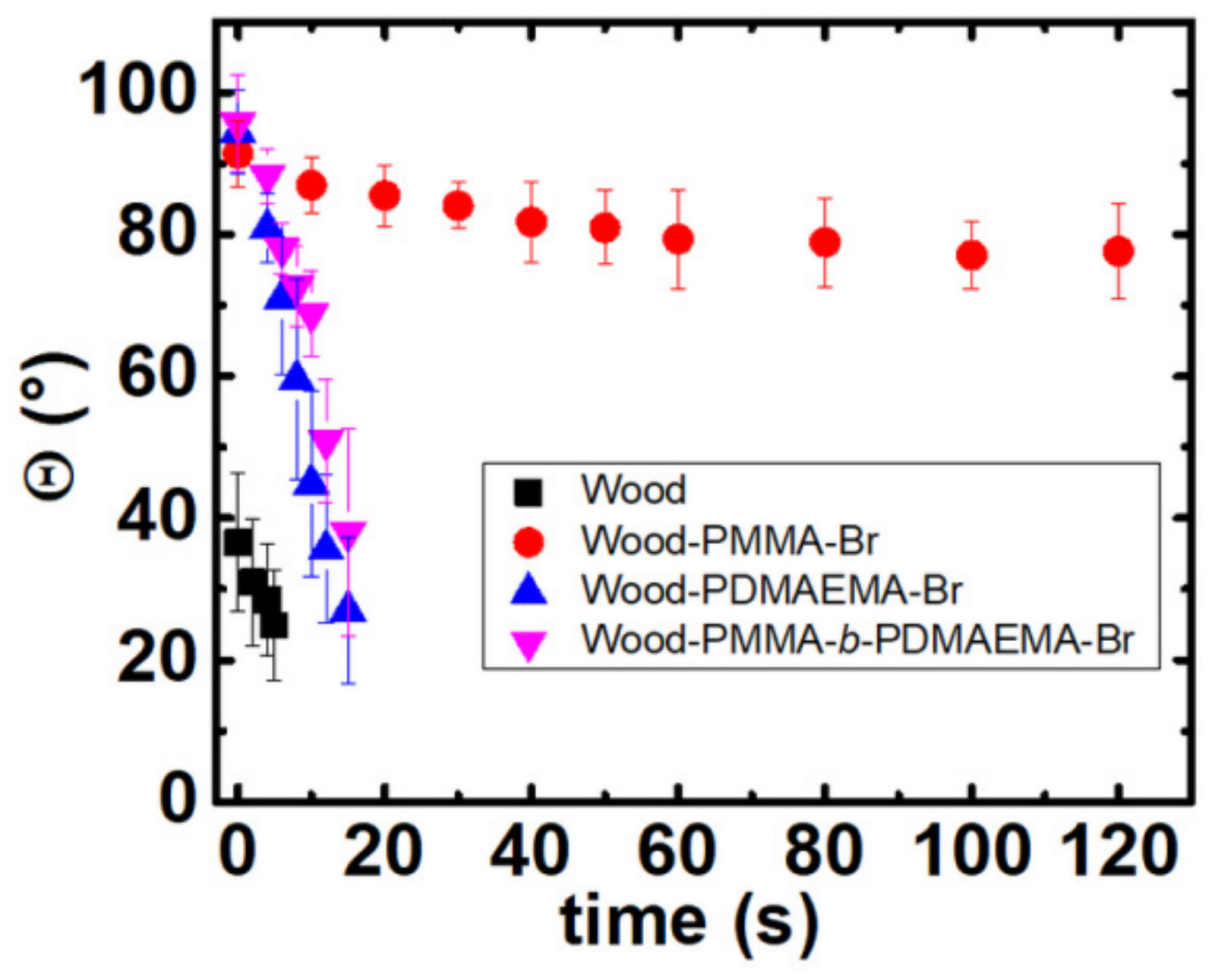
Contact angle measurements for unmodified wood and wood modified with polymers at 22 °C during 120 s, PMMA (72 ppm by wt), PDMAEMA (45 ppm by wt) and PMMA-*b*-PDMAEMA copolymers.

The general effect of wood fiber modifications through the grafting of PMMA is to increase its water repellency. To evaluate the hydrophilic/hydrophobic properties of the wood and modified wood cubes with PMMA and PDMAEMA, the water contact angle was examined by the sessile drop method at room temperature (Figure 6). To further illustrate the changes in water absorption, polymers were synthesized at different catalyst concentrations (see details in Supplementary Materials: Section S7). The results show that the properties of the wood surface can be adjusted with the proposed method.

The native wood is characterized by high hydrophilicity due to the presence of hydroxyl groups in its structure, and the initial water contact angle was Θ~36° (Figure 6), and a drop of water soaked into the wood surface after 10 s. In wood grafted with copolymer, the PMMA is covered by a PDMAEMA layer, so the water seeps into the first layer but stays on the second one, therefore, it does not penetrate the wood. The modification of the wood surface with hydrophobic PMMA increased the value of the contact angle to Θ~92° with very little change in soaking during 120 s of exposure. PDMAEMA modified wood showed a bigger water absorption than in the presence of a hydrophobic polymer. A change in the surface tension of the water may explain this observation. Surface tension measurements have shown that these copolymers have specific surface activities and can reduce the surface tension of water [54,55].

**Figure 7:**
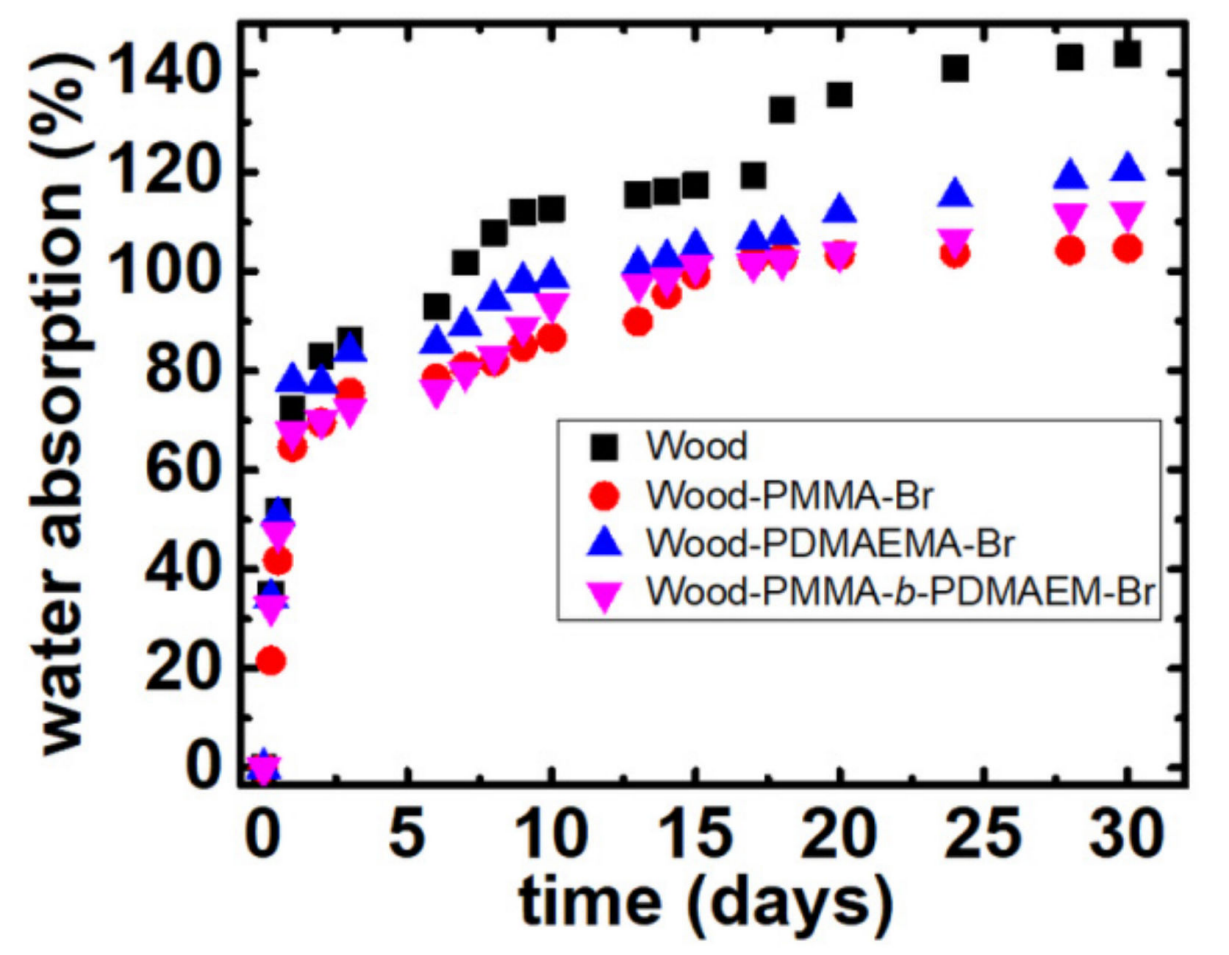
Water absorption results of untreated wood and wood–polymer composites–values shown for PMMA (72 ppm by wt), PDMAEMA (45 ppm by wt) and PMMA-*b*-PDEAEMA copolymers.

The water absorption tests of native and polymer modified wood were also carried out, showing the relationship between water absorption and immersion time. The water absorption values of the unmodified wood sample showed an increase from 51% (after 6 h) to 144% after 30 days of exposure to water. Water absorption values decreased after the polymer modification (Figure 7).

The PMMA modified wood showed reduced absorption properties, absorbing 22% and 105% water after 6 h and 30 days, respectively. The addition of the PDMAEMA polymer block increased the water uptake (32% after 6 h to 112% after 30 days). It is related to the attachment of a hydrophilic polymer brush block. Samples coated with PDMAEMA absorbed water slightly faster than PMMA modified wood–from 34% of water uptake after 6 h to 120% after 30 days.

**Figure 8:**
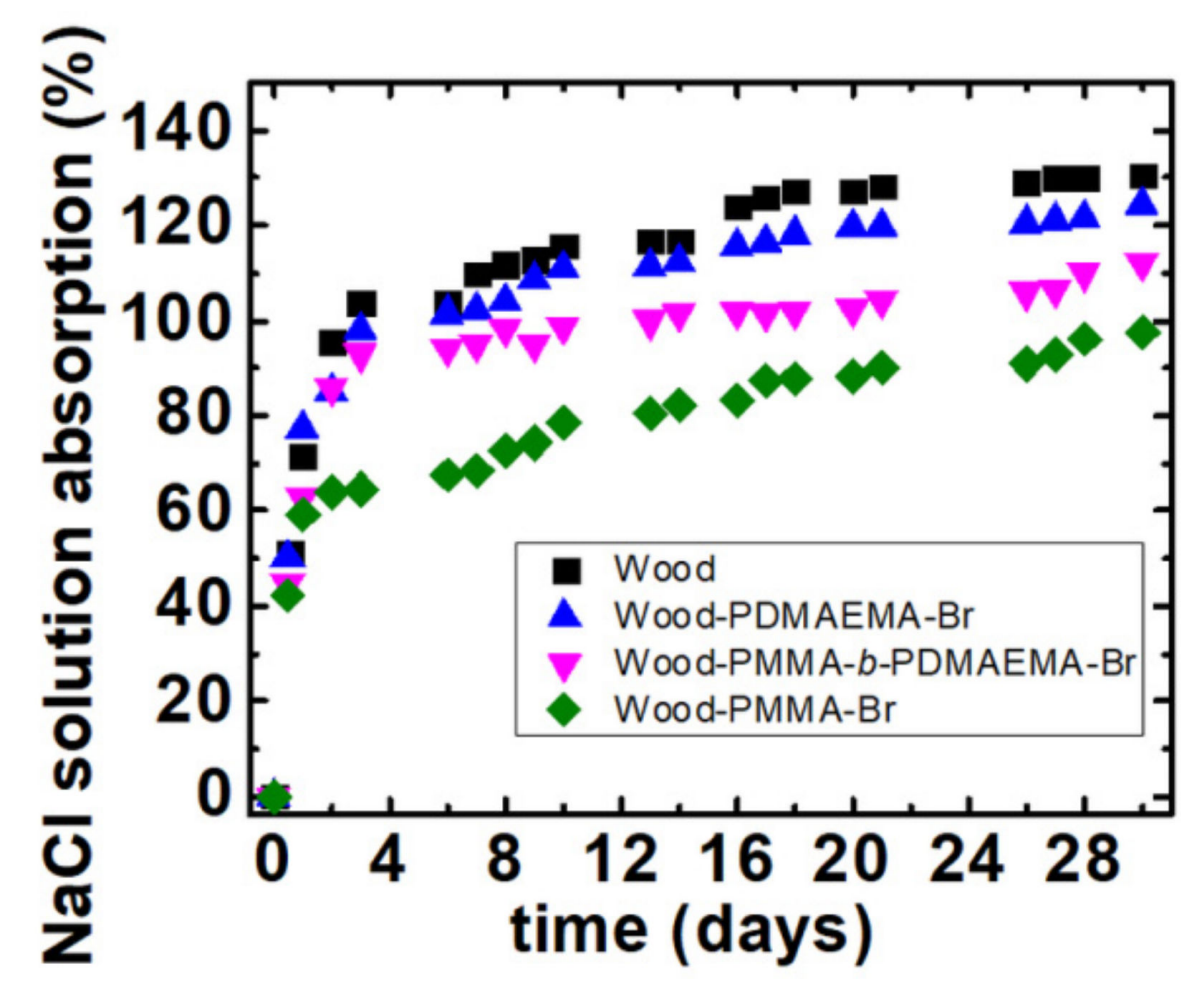
Aqueous NaCl solution absorption results of untreated wood and wood–polymer composites values shown for PMMA (273 ppm by wt), PDMAEMA (45 ppm by wt) and PMMA-*b*-PDEAEMA copolymers (polymer block consisting of PMMA—72 ppm by wt and PDMAEMA—45 ppm by wt).

Polymer modified wood has potential applications in marine, civil, and aviation engineering, and is also suitable for use in humid places such as bathroom interiors, wooden terraces, food packaging [56,57]. For this reason, the obtained samples of polymer modified wood were immersed in an aqueous salt solution imitating the salinity level in the Baltic Sea—7‰ [58] (Figure 8).

Untreated wood showed the high absorption of NaCl solution—51% after 12 h, up to 130% after 30 days of exposure. The PMMA modified wood showed reduced absorption properties, absorbing 42% and 98% saline after 12 h and 30 days, respectively. On the other hand, the samples modified with the PMMA-*b*-PDMAEMA copolymer showed absorption of only 45% after 12 h to 112% until the end of the study. Raw wood is characterized by high hydrophilicity due to the presence of ubiquitous hydroxyl groups in its structure. The PMMA modified wood blocks showed water repellent properties and lower absorption of NaCl solution. Samples coated with PMMA-*b*-PDMAEMA gave a wood–polymer hybrid material with weaker hydrophobic properties. This phenomenon is caused by the coating of the PDMAEMA hydrophilic layer, which caused a slight increase in the absorption of the aqueous NaCl solution. The results show that the samples of wood modified with copolymers are resistant to the absorption of moisture and salt solutions. A significant difference was observed between the biocomposites and the control samples because the unmodified wood showed much higher water and NaCl absorption values than the modified samples.

**Figure 9:**
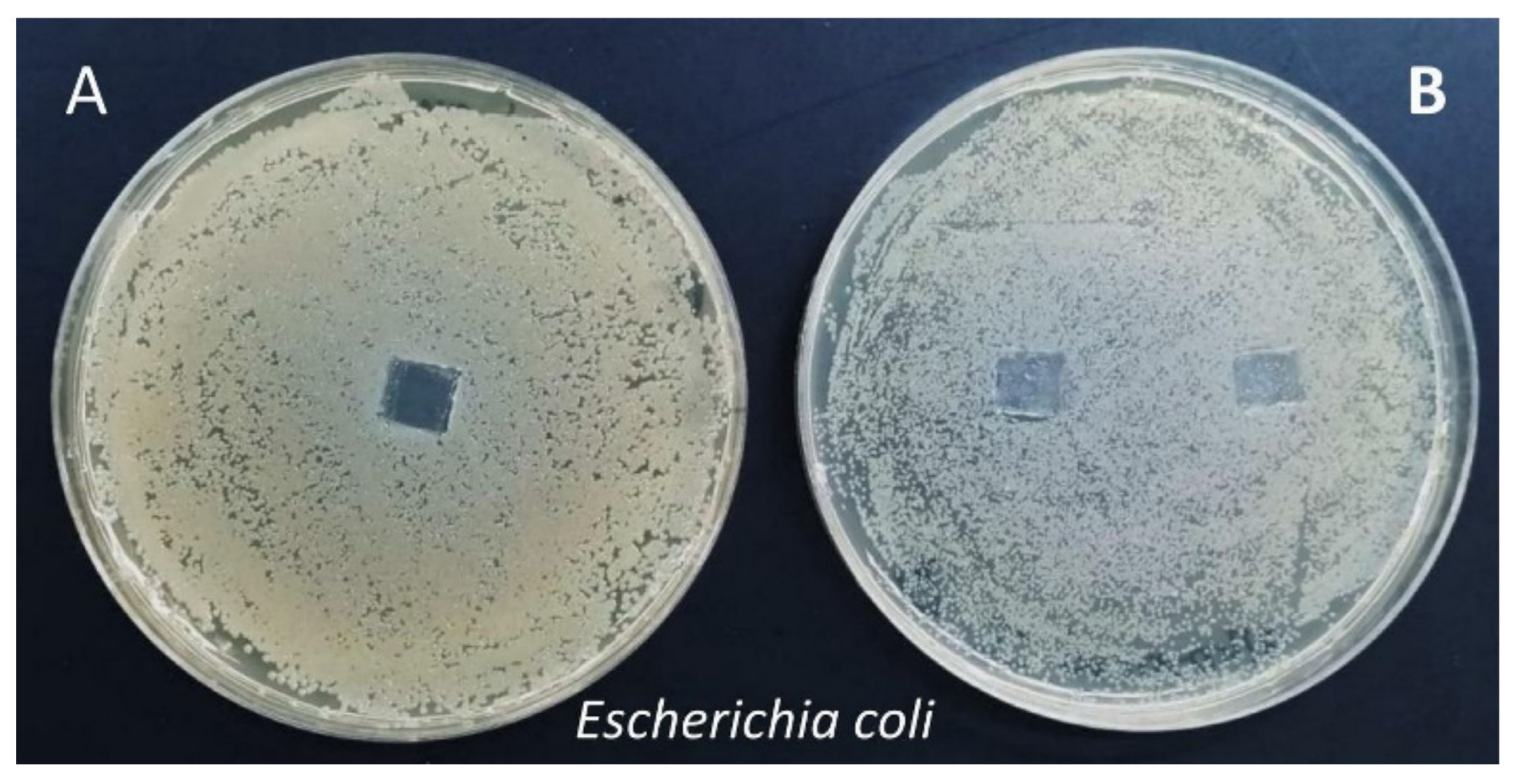
Antibacterial activity identified by agar diffusion method of wood-QPDMAEMA (**A**) in comparison with unmodified wood (**B**) against Gram-negative *Escherichia coli*.

Two different representative strains were adopted, a Gram-negative (*E. coli*) and Gram-positive (*S. aureus*), which are the main human bacterial pathogens causing a wide variety of clinical symptoms, were used to evaluate antimicrobial properties of modified wood after PDMAEMA block quaternization (wood-QPDMAEMA-Br and wood-PMMA-*b*-QPDMAEMA-Br). The agar plate diffusion assay revealed minimal antibacterial properties, as no inhibition zone was observed. However, no bacterial growth was observed in the place of the direct contact of the wood blocks (Figure 9A) with the *S. aureus* and *E. coli* inoculum (for modified wood blocks) in comparison with the presence of bacteria in unmodified control (Figure 9B).

**Figure 10:**
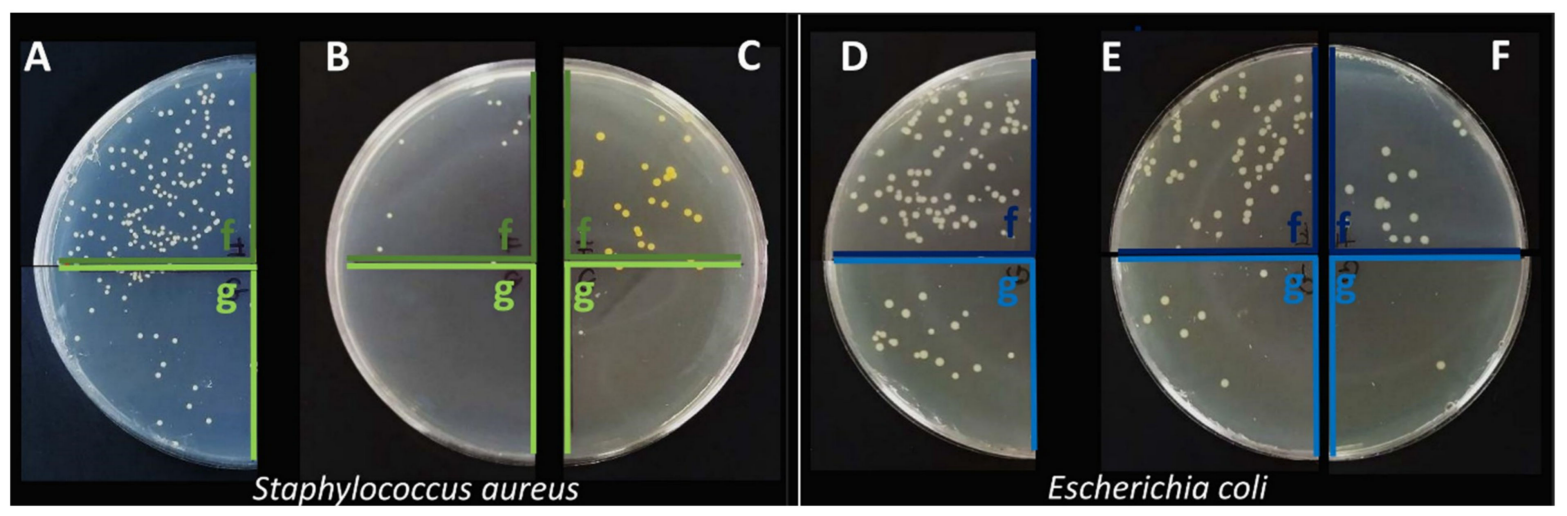
Antibacterial activity identified by plate counting method with serial dilutions of *S. aureus* and *E. coli* cultures recovered from: (**A**,**D**) control wood block, (**B**,**E**) wood-QPDMAEMA and (**C**,**F**) wood-PMMA-*b*-QPDMAEMA; f and g are, 10^5^- and 10^6^-fold dilutions of recovered bacterial culture, respectively.

The organic and porous nature of the wood makes it a difficult object for research, as the bacteria penetrate the pores and may not come out after simple rinsing of tested wood blocks [59]. To recover the highest concentration of bacteria, we used the elusion dependent method, where the contaminated tested wood blocks were directly immersed in a sterilized eluent (PBS) and then a physical dissociation by vortexing was performed. Preparing serial dilutions of recovered bacteria allowed the assessment of antibacterial properties of wood-PMMA-*b*-QPDMAEMA-Br and wood-QPDMAEMA-Br. The performance of the plate counting method [60] with serial dilutions made it possible to calculate several indicators (survival fraction, percentage of reduction and log reduction, SR, LR, and PR, respectively) determining the antibacterial properties (Table 4, Figure 10).

**Figure 11:**
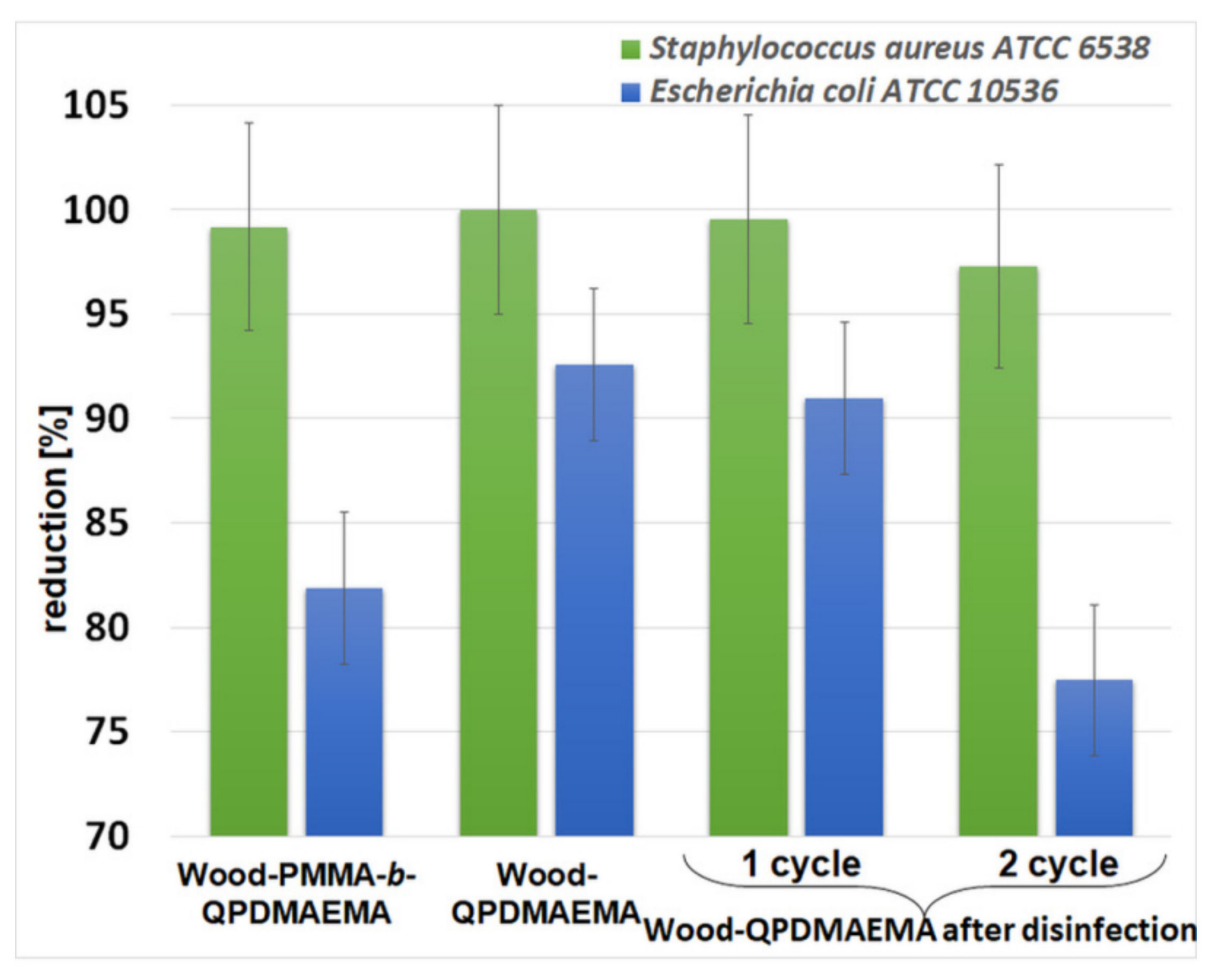
Percent of reduction (PR) in *S. aureus* and *E. coli* culture density after incubation on modified wood blocks in reference to control wood sample.

The log reduction of CFU/mL counts greater than 2 are considered as a bactericidal effect [60,61], thus, the results of wood-QPDMAEMA-Br (≈ 4log or 99.99% of effective bactericidal activity) indicate its strong bacteria-killing ability. The highest value of the reduction of *S. aureus* cell number was observed for wood-QPDMAEMA-Br (99.997%), which makes only 0.003% of bacteria survive after 24 h contact with a tested wood block. A more resistant *E. coli* strain shows a lower percentage of bacterial growth reduction, however, same as for *S. aureus*, it is higher for wood-QPDMAEMA in comparison with wood-PMMA-*b*-QPDMAEMA-Br (92.58% vs. 81.88%). Figure 11 represents the comparison of antibacterial activity of modified wood against *S. aureus* and *E. coli* strains.

Interestingly, after the thorough disinfection of wood-QPDMAEMA-Br block by immersion in ethanol and exposure to UV radiation, the survival fraction of *S. aureus* and *E. coli* cells was higher, thus, more bacterial cells survive incubation with modified wood blocks. A decrease (by 3% and 15%, respectively, against *S. aureus* and *E. coli*) in the antibacterial capacity of the modified wood-QPDMAEMA-Br block, depending on the number of sterilization cycles, was also observed, which seems likely due to the greater susceptibility of *S. aureus* to the tested modified wood blocks.

Reaction solution with 3.39 mg/mL of QPDMAEMA (quaternized poly(2-(dimethylamino)ethyl methacrylate) was also found to have antibacterial activity, both against Gram-positive and Gram-negative strains (Table 5). The higher minimal inhibitory concentration/minimal bactericidal concentration (MIC/MBC) value for *E. coli* confirms the greater resistance of this strain and indicates that, in the range between 52.97 and 423.8 µg/mL, it has a bactericidal effect on *E. coli*. Interestingly, MIC concentration against *E. coli* is twofold lower than that of oxacillin, a beta-lactam antibiotic with documented in vitro activity against Gram-negative aerobic bacteria [62]. A lower MIC concentration of QPDMAEMA against *S. aureus* indicates a higher susceptibility of Gram-positive bacteria. These outcomes confirm the results for modified wood blocks. Live bacteria adhere very strongly to QPDMAEMA surfaces due to the formation of electrostatic interactions [60], which prevents their desorption even after intensive washing of the polymer. However, the application of appropriate controls, without the potentially antibacterial factor, enables the comparison of activity under real conditions, without the need to destroy the coatings.

It can be noticed that, generally, *E. coli* is more resistant to the presence of QPDMAEMA in the environment, both in the form of a solution (Table 5) and in the form of a coating on wood blocks (Table 4). These results are consistent with those of other authors, as *E. coli* has a double cellular membrane that may complicate the polymer interaction, resulting in slower cell death in comparison with *S. aureus* [60,63].

## 3. Experimental

### 3.1. Chemicals

Ash wood (*Fraxinus excelsior*) was cut into samples of dimensions 8 mm × 8 mm × 5 mm (longitudinal × tangential × radial) and extracted using a benzene–ethanol (EtOH) mixture (2:1 *v*/*v*). In addition, 2-bromoisobutyryl bromide (BriBBr, 98%, Sigma Aldrich, St. Louis, MO, USA), dichloromethane (DCM, >99.9%, Sigma Aldrich), pyridine (99%, Sigma Aldrich)*,* tetrahydrofuran (THF, >99%, Sigma Aldrich), ethyl 2-bromoisobutyrate (EBiB, 98%, Sigma Aldrich), copper(II) bromide (Cu^II^Br_2_, 99.9%, Sigma Aldrich), benzene (>99.7%, Chempur, Piekary Slaskie, Poland), methanol (>99.8%, Chempur), acetone (>99.5%, Stanlab, Lublin, Poland), ethanol (EtOH, ≥99.8%, Honeywell Riedel-de Haën, Morristown, NJ, USA), *N,N*-dimethylformamide (DMF, 99.9%, Acros, Fair Lawn, NJ, USA), *N*,*N*-dimethylformamide (DMF CHROMASOLV™, ≥99.9%, GPC analysis, Honeywell Riedel-de Haën), bromoethane (98%, Sigma Aldrich), were not subjected to further purification. Tris(2-pyridylmethyl)amine (TPMA) and Cu^II^Br_2_/TPMA stock solutions were prepared as described elsewhere [64]. Methyl methacrylate (MMA, >99%, Sigma Aldrich) and 2-(dimethylamino)ethyl methacrylate (DMAEMA; 98%, Aldrich) was passed through a column filled with basic alumina prior to use to remove any inhibitor [65]. Ag^0^ wire (99.9%, Alfa Aesar, Kandel, Germany) was purified with an aqueous solution of NaCl at 95 °C and washed with acetone. Brominated silicon wafers were synthesized according to previously reported procedure [66]. Bacterial representative strains, media, and culture conditions: Escherichia coli ATCC 10536 (*E. coli*) and Staphylococcus aureus ATCC 6538 (*S. aureus*) were used as a part of collection of Department of Biotechnology and Bioinformatics, Faculty of Chemistry, Rzeszow University of Technology, Poland. Mueller Hinton Broth (MHB, NutriSelect^®^ Plus, pH 7.4), Mueller Hinton Agar (MHA, NutriSelect^®^ Plus, pH 7.3), phosphate buffered saline (pH 7.4), oxacillin sodium salt monohydrate (OXA, >97%) and rifampicin (RIF, ≥97%) were purchased from Sigma Aldrich St. Louis, MO, USA. Tetracycline hydrochloride (TET, ≥95%) and gentamycin sulphate (GEN) were purchased from Carl Roth GmbH+ Co., Karlsruhe, Germany. Both bacterial strains were grown from frozen stocks and subcultured at least twice before use in experiments to ensure normal growth patterns. The method of preparing the initial bacterial culture was the same for all used antibacterial activity assays. Briefly, after 24 h of incubation at 37 °C in New Brunswick Innova 40 Shaker (Eppendorf AG, Hamburg, Germany), the number of cells in suspension was adjusted to the 0.5 McFarland standard (10^8^ colony-forming units, CFU/mL; OD_600_ = 0.46) with the use BIO-RAD SmartSpec^TM^ Plus Spectrophotometer (Hercules, CA, USA). All reagents and bacterial cultures were prepared using Laminar Flow Cabinet ESCO Airstream (Esco Lifesciences GmbH, Friedberg, Germany).

### 3.2. Analysis

#### 3.2.1. Proton Nuclear Magnetic Resonance Spectroscopy (^1^H NMR)

The polymerization kinetics investigation was performed by ^1^H NMR analysis of the samples taken from the solution. Analysis was carried out in CDCl_3_ using Bruker Avance 500 MHz spectrometer (Bruker, Karlsruhe, Germany) in 25 °C.

#### 3.2.2. Gel Permeation Chromatography (GPC)

Size exclusion chromatography (GPC) was performed to determine number average molecular weights and molecular weight distributions using Shimadzu modular system (Kyoto, Japan) comprising of a CBM-40 system controller, RID-20A differential refractive-index detector, a SIL-20AHT automatic injector, one precolumn and three PSS GRAM (PSS Polymer, Mainz, Germany) 10μm columns, pore size: one 100 Å column and two 3000 Å columns. The temperature of the columns was maintained at 35 °C by a CTO-20A oven. The eluent in chromatographic separation was *N*,*N*-dimethylformamide (HPLC grade, with 0.01 M LiCl) with the flow rate was kept at 1 mL min^−1^ using an LC-40 pump. A molecular weight calibration curve was generated from polystyrene standards with narrow molecular weight distribution (PSS Polymer Standards Service, Mainz, Germany).

#### 3.2.3. Fourier Transform Infrared Spectroscopy (FT-IR)

The chemical structure of the wood surface (unmodified wood), brominated wood, and wood–polymer composites were analyzed by spectrophotometer Nicolet 6700 FT-IR (Thermo Scientific, Waltham, MA, USA), within 500–4000 cm^−1^, with the use of attenuated total reflectance (ATR) technique.

#### 3.2.4. Scanning Electron Microscopy (SEM)

The polymer coated wooden cubes were visualized with a Phenom Pro Desktop Scanning Electron Microscope (Thermo Fisher, Waltham, MA, USA). The acceleration voltage of 10 kV was used for measurements.

#### 3.2.5. Atomic Force Microscopy (AFM)

The topography images of the synthesized polymer layers on silicon substrates were measured by Dimension Icon AFM microscope (Bruker, Santa Barbara, CA, USA) operating in the Peak Force Tapping (PFT) and QNM^®^ modes with standard silicon cantilevers with a nominal spring constant of 0.4 N/m. The thicknesses were determined by making the scratch (by metal tweezers) on the polymer layer and capturing the AFM image (resolution 256 × 256 pixels) at the edge of the scratch. The thicknesses were calculated from the depth profiles as a difference in height between the polymer layer and uncovered silicon substrate. Before the measurements, the scratched brushes were sonicated in organic solvents in order to remove any loosely bound polymer.

#### 3.2.6. Water Contact Angle Measurements

The hydrophobicity of the polymer layer grafted from ash wood samples was measured by determining the values of contact angles. The values of the contact angles were obtained from the geometrical analysis of the photos taken for three drops (10 µL) of water applied to the wood samples (temperature 22 °C). The results were then analyzed by the Kropla program to interpret Young’s equation in line with our previous research [65]. Pictures were taken with a Panasonic DC-FZ82 camera equipped with a LUMIX DC VARIO 1: 2.8–5.9/3.58–215 ASPH lens.

#### 3.2.7. Antibacterial Activity Assays

To evaluate the antimicrobial properties of the modified wood-QPDMAEMA and wood-PMMA-*b*-QPDMAEMA surfaces, two different representative strains of bacteria were used in the research: gram-negative (*E. coli*) and gram-positive (*S. aureus*). The antibacterial activity of modified (wood-PMMA-*b*-QPDMAEMA-Br and wood-QPDMAEMA-Br) and nonmodified control wood block was evaluated by the bacterial inhibition ring method (agar plate diffusion test CEN/TC 248 WG 13) [67], followed by modified bacterial growth reduction assay (EN ISO 20743:2007 Transfer Method) [68] against the Gram-positive S. aureus and Gram-negative E. coli. Minimum inhibitory concentration and minimum bactericidal concentration evaluation methods [69] were performed to verify the antibacterial properties of Q-PDMAEMA solution, used to modify wood surfaces.

#### 3.2.8. Antibacterial activity of modified wood

The evaluation of the antibacterial activity of modified wood blocks was as follows. MHA was prepared in distilled water and autoclaved. The agar medium was then poured on sterile Petri dishes (Ø 90 mm), cooled out, and inoculated with 10^5^ CFU/mL of *S. aureus* culture (1000-times diluted initial bacterial culture). After UV treatment, each wood (modified and control sample) was planted onto the agar plates and incubated at 37 °C for 24 h, transferred into sterile PBS and vortexed (Scilogex MX-S, Rocky Hill, USA) for 90 s in order to recover the maximum amount of bacteria that penetrated through the wood pores. Ten-fold serial dilutions of each recovered bacterial suspension were prepared in MHB. MHA plates were inoculated with the obtained dilutions and incubated for 24 h at 37 °C. Then, colony counting was performed manually. The rate of bacteria removed from wood-PMMA-*b*-QPDMAEMA and wood-QPDMAEMA was related to the amount of bacteria recovered from the control wood. Efficacy of particular wood blocks in reduction of viable bacteria number was calculated by comparing the density of viable microbes on modified to the density on unmodified samples. The survival fraction (SF), percentage of reduction (PR) and log reduction (LR) after 24 h of exposure on wood samples were calculated with the use of Equation (1), Equation (2) and Equation (3), respectively:

(1) $$\mathrm{SF}=\frac{\mathrm{number}\;\mathrm{of}\;\mathrm{CFU}/\mathrm{mL}\;\mathrm{after}\;\mathrm{exposure}}{\mathrm{number}\;\mathrm{of}\;\mathrm{CFU}/\mathrm{mL}\;\mathrm{in}\;\mathrm{control}}$$

(2) $$\mathrm{PR}=\left(1-\mathrm{SF}\right)\times 100\%$$

The LR value is the reciprocal of SF after transformation to a logarithmic scale (3):

(3) $$\mathrm{LR}=\log_{10}\left({\mathrm{SF}}^{-1}\right)$$

In order to evaluate the antibacterial effectiveness of wood-PMMA-*b*-QPDMAEMA-Br and wood-QPDMAEMA-Br exposed to washing and decontamination, two cycles of disinfection were carried out. Each cycle consisted of 30 min soaking of wood block in 70% ethanol, air drying, and UV sterilization (all activities were carried out under Laminar Flow Cabinet ESCO Airstream in order to ensure aseptic conditions). After each cycle, the antibacterial activity assays of wood-PMMA-*b*-QPDMAEMA-Br, wood-QPDMAEMA-Br, and control wood were repeated.

#### 3.2.9. Minimum Inhibitory/Minimum Bactericidal Concentration (MIC/MBC)

Minimum inhibitory and bactericidal concentrations were determined in order to verify the antibacterial properties of reaction solution containing QPDMAEMA, in which modified wood samples (wood-PMMA-*b*-QPDMAEMA-Br and wood-QPDMAEMA-Br) were prepared. Series of two-fold dilutions were prepared on a 96-well plate in MHB. An appropriate bacterial culture at 10^5^ CFU/mL density was added to the prepared series of solution dilutions and incubated at 37 °C. After 24 h, bacterial growth in comparison with the positive control (the medium without antibacterial agents) was monitored. The MIC was defined as the lowest concentration of the antibacterial agent, which completely inhibited the visible growth of the microorganism. These results were confirmed by measurement of the optical density (OD_600_) at 600 nm (BIO-RAD Model 550 Microplate Reader, Hercules, CA, USA). The experiment was carried out in triplicate. A positive (the medium without antibacterial agents) and negative control (no bacterial cultures added) of bacterial growth and solvent control were performed. An evaluation of the antibiotic susceptibility of both strains to tetracycline (TET), gentamicin (GEN), oxacillin (OXA) and rifampicin (RIF) was also performed by the microdilution method (range from 0.03 to 500 µg/mL for TET, GEN and OXA; range from 0.03 to 125 µg/mL for RIF).

#### 3.2.10. Statistical Analysis

For each assay, two samples of wood-PMMA-*b*-QPDMAEMA-Br and wood-QPDMAEMA-Br, and two nonmodified control wood samples were analyzed. Mean colony counts and standard deviation (SD) were calculated for each wood sample and controls. A *p*-value < 0.05 was considered statistically significant. Statistical analyses were performed using STATISTICA v.12 software (StatSoft, OK, USA).

### 3.3. Immobilization of BriBBr on Ash Wood Surface (Wood-Br)

The wood samples were extracted with a Soxhlet apparatus with a benzene–ethanol mixture (100 mL/50 mL; 2:1) for 8 h and then dried under vacuum (80 °C, −62 kPa) for 24 h. The samples were put into a 10 mL Schlenk flask equipped with a condenser tube, BriBBr as initiator precursor, pyridine in a molar ratio to the –OH groups of wood (–OH:BriBBr:pyridine = 1:1:1) (see details in Supplementary Materials: Section S1). The calculated amount of BriBBr, pyridine, and CH_2_Cl_2_ (4 mL) was added to the flask. Then, wooden cubes were introduced and placed on poles in rubber partitions. The reaction was run under an inert N_2_ gas atmosphere with stirring at 700 rpm and heated to 58 °C for 1 h. The wood sample was then removed from the reaction mixture and thoroughly washed with a stream of CH_2_Cl_2_ and THF and washed in an ultrasonic bath with CH_2_Cl_2_ and THF (10 min each). Extraction with ethanol was performed for 5 h to completely remove unreacted starting materials. The extracted samples were placed successively in a vacuum oven (80 °C, −62 kPa) for 24 h. The obtained product was characterized by FT-IR analysis and abbreviated as wood-Br.

### 3.4. General Procedure for Ag^0^ SI-ARGET ATRP of MMA from a Wood Surfaces

The brominated wood samples were placed in a jacketed five necked flask equipped with a magnetic stirrer. The reaction mixture contained: MMA monomer (6.04 mL, 0.057 mol), EBiB (14 μL, 0.09 mmol), 0.05 M Cu^II^Br_2_/TPMA stock solution (0.340 μL, 0.226 μL, 0.113 μL, 0.091 μL and 0.011 μL 0.05 M in DMF) and DMF to obtain 15 mL of total reaction volume. Then the Ag^0^ wire (dimension = 80 cm long; thickness 0.05 mm), previously purified with an aqueous solution of NaCl at 95 °C and washed with acetone, was placed in a rubber septum placed in one neck of the flask. The flask was degassed with argon for 45 min and the reaction mixture was heated to 50°C using a laboratory thermostat (Labo Play ESM-3711-H). The polymerization reaction was started after the Ag^0^ wire was added to the reaction mixture. Samples were taken periodically to follow the monomer conversion by ^1^H NMR analysis and to check the number average molecular weight (*M*_n_) and the dispersion index (*M*_w_/*M*_n_) of the linear polymers grown from the sacrificial initiator using GPC analysis. Before GPC analysis, the polymer samples were dissolved in DMF + 0.01 M LiCl + toluene as an external standard–mobile phase and passed through a neutral alumina column with a 0.22 µm syringe filter to remove the catalyst. The polymerization reaction was stopped by opening the flask and exposing the catalyst to air. The obtained wood–polymer composites were cleaned by ultrasonication for 3 min in THF and DCM. To completely remove unreacted substrates from the wood samples, Soxhlet extraction with ethanol was performed for 7 h and the samples were dried under vacuum (60 °C, −62 kPa) for 24 h. For characterization of the final linear polymer product, purification was carried out by precipitation in water/methanol mixture, then dried under vacuum and analyzed by ^1^H NMR.

### 3.5. General Procedure for SI-ARGET ATRP of DMAEMA from a Wood Surfaces

The brominated wood samples and two silicon wafers were placed in a five neck jacketed flask equipped with a magnetic stirrer. The first reaction mixture contained: DMAEMA monomer (5.96 mL, 0.035 mol), EBiB (9 μL, 0.06 mmol), Cu^II^Br_2_/TPMA stock solution (57 μL of 0.05 M in DMF), and DMF to give a total reaction volume of 15 mL. Then, an Ag^0^ wire (dimension = 80 cm long; thickness 0.05 mm), previously purified with an aqueous NaCl solution at 95 °C and washed with acetone, was placed in the rubber septum placed in one neck of the flask. The second reaction mixture contained: DMAEMA monomer (5.96 mL, 0.035 mol), EBiB (9 μL, 0.06 mmol), Cu^II^Br_2_/TPMA stock solution (57 μL of 0.05 M in DMF), and DMF to give a total reaction volume of 15 mL. The third reaction mixture contained: DMAEMA monomer (8.03 mL, 0.048 mol), EBiB (12 μL, 0.08 mmol), Cu^II^Br_2_/TPMA stock solution (95 μL 0.05 M in DMF), and DMF to give a total reaction volume of 20 mL. All flasks were degassed with argon for 45 min and the reaction mixture was heated to 50 °C with a laboratory thermostat (Labo Play ESM-3711-H). All polymerization reactions started after the addition of the Cu^II^Br_2_/TPMA complex which was introduced after degassing the solution. The completion of the polymerization reaction and its progress control was analogous to that in procedure 3.4.

### 3.6. Measurements of Water and NaCl Solution Absorption

The weight of the wood cubes after polymerization was determined by weighing on an analytical balance. Then, the samples were immersed in distilled water or NaCl salt solution (7‰) in a glass beaker. The samples were taken at specified time intervals, wiped from adhering solvents, and weighed on an analytical balance. Tests were performed at room temperature. The percentage absorption (*A*) was calculated by the Equation (4):

(4) $$A=\frac{m_{t}-m_{0}}{m_{0}}$$

where, *m*_t_ is the mass of sample at time *t* and *m*_0_ is the dry weight of sample.

### 3.7. Quaternization of PDMAEMA Grafted from the Wood Surface

The conditions of the quaternization reaction were determined in relation to the increase in the PDMAEMA polymer mass grafted from the surface of the wood (wood-PDMAEMA-Br and wood-PMMA-*b*-PDMAEMA-Br). For the quaternization of PDMAEMA on the wood surface, 15 mL of acetone was used as a solvent and bromoethane (bromoethane: amino groups in DMAEMA units in a molar ratio of 35:1).The reaction was carried out in a flask under atmospheric conditions. Wood samples were introduced into the reaction mixture using tourniquets. The flask was placed in an oil bath at 40 °C and the mixture was held on a magnetic stirrer with stirring at 700 rpm for 96 h. After this time, the mixture became straw-colored. Then the acetone was decanted, and the wood was washed for 3 min with acetone using ultrasound [70].

### 3.8. Quaternization of PDMAEMA from Solution

The quaternization reaction conditions were determined with respect to the weight of the PDMAEMA polymer. For the quaternization of PDMAEMA on the wood surface, 10 mL of acetone was used as solvent and bromoethane (bromoethane: amino groups in a molar ratio of 35:1 molar excess to DMAEMA units). The reaction was carried out in a flask under atmospheric conditions. The flask was placed in an oil bath at 40 °C and the mixture was kept on a magnetic stirrer with stirring at 700 rpm for 96 h.

## 4. Conclusions

This work presents the use of the SI-ARGET ATRP concept with Ag^0^ wire to polymerize MMA from wood cubes, while during the polymerization of DMAEMA no additional reducing agent was used, due to self reducing properties of the monomer. The two-step procedure consisting of a bromination step followed by surface initiated ARGET ATRP with low catalyst loading was performed.The results were visualized by scanning electron microscopy (SEM) to compare the structure of untreated, brominated and polymer-coated wood cubes indicateing that PMMA and PDMAEMA were indeed inoculated on the wood surface. After polymer modification, the polymer coated wood showed a rough texture with full coverage with no visible structural elements of the wood cell walls. It is visible especially on the high magnification SEM micrographs of modified wood. This is in line with the results of the FT-IR analysis, namely, there are a clear difference in the unmodified wood structure and modified wood, both with initiation sites and polymers. The water contact angles of the PMMA grafted wood surface were significantly affected by the amount of polymer covering the surface controlled by the amount of catalyst concentration. Excellent water repellent properties and longer wood surface stability (ranged from Θ ~ 95°) were obtained without notable changes after 120 s of water droplet exposure. Absorption tests confirmed a significant improvement in the hydrophobic properties of wood samples–samples coated with polymers showed a lower absorption of water and saline water (7‰) compared to untreated wood. Water absorption tests confirmed a significant improvement in the properties of ash wood samples-samples modified with polymers showed lower absorption of water and NaCl solutions compared to untreated wood. Antibacterial tests revealed that wood-QPDMAEMA-Br, as well as wood-PMMA-*b*-QPDMAEMA-Br, exhibit higher antibacterial activity against Gram-positive *Staphylococcus aureus* in comparison with Gram-negative *Escherichia coli,* both as a solution and as a coating on wooden blocks.

Wood fibers grafted in PMMA and PDMAEMA block can act as multifunctional wood-polymer composites with excellent hydrophobic and antibacterial properties. Based on these results, it can be concluded that these composites are potentially suitable for use in wet rooms, such as bathroom interiors, wooden decks, food packaging, where bactericidal properties are important. A controlled radical polymerization technique has proven useful in the synthesis of functional macromolecules with a controlled and complex architecture on the surface of the wood.

## Figures and Tables

**Table 1:** Ag^0^ SI-ARGET ATRP of MMA with different catalyst loadings ^1^.

Entry	[MMA]_0_/[EBiB]_0_/[Cu^II^Br_2_/TPMA]_0_	[Cu^II^Br_2_]_0_, (ppm by wt)	Conv ^2^ (%)	*k*_p_^app 3^ (h^−1^)	DP_n_,_theo_^2^ (per chain)	*M*_n,theo_^4^ (×10^−3^)	*M*_n,app_^5^ (×10^−3^)	*M*_w_/*M*_n_^5^
1	600/1/0.180	273	67	0.124	400	40.2	21.6	1.44
2	600/1/0.120	182	60	0.088 0.087 ^6^	362	36.5	22.2	1.43
3	600/1/0.060	90	62	0.101 0.104 ^6^	370	37.2	20.6	1.33
4	600/1/0.048	72	71	0.120	426	42.8	17.9	1.31
5	600/1/0.006	9	69	0.110 0.108 ^6^	415	41.7	17.8	2.08

^1^ General conditions: T = 50 °C; *V*_tot_ = 15 mL; *t* = 9.5 h for entry 1, *t* = 10 h for entry 2, *t* = 10.25 h for entry 3, *t* = 9.5 h for entry 4, *t* = 10.25 h for entry 5. SI-ARGET ATRP with silver wire: *d* = 0.05 cm, *l* = 80 cm; ^2^ Monomer conversion (Conv) and theoretical degree of polymerization (DP_n_,_theo_) calculated according to ^1^H NMR analysis of the samples withdrawn from the solution, DP_n_,_theo_ = (Conv · [MMA]_0_)/[EBiB]_0_; ^3^ Apparent rate constant of propagation, calculated as a slope of the curve ln([M]_0_/[M]) = *f(t*) (illustrated in the Figure 1a); ^4^ *M*_n,theo_ = ([MMA]_0_/[EBiB]_0_) Conv *M*_MMA_ + *M*_EBiB_; ^5^ *M*_n,app_ and *M*_w_/*M*_n_ were determined by DMF GPC; ^6^ Apparent rate constant of propagation for polymerization time of 9.5 h. Abbreviations: Ag^0^ SI-ARGET ATRP surface initiated activators regenerated by electron transfer atom transfer radical polymerization mediated with Ag^0^ as a reducing agent, MMA—methyl methacrylate, Cu^II^Br_2_/TPMA—copper(II) bromide/tris(2-pyridylmethyl)amine catalytic complex, EBiB—ethyl 2-bromoisobutyrate.

**Table 2:** Polymerization of DMAEMA by internally controlled SI-ARGET ATRP concept ^1^.

Entry	[DMAEMA]_0_/ [EBiB]_0_/[Cu^II^Br_2_/TPMA]_0_	[Cu^II^Br_2_]_0_, (ppm by wt)	Reducing Agent	Conv ^2^ (%)	*k*_p_^app 3^ (h^−1^)	DP_n_,_theo_^2^ (per chain)	*M*_n,theo_^4^ (×10^−3^)	*M*_n,app_^5^ (×10^−3^)	*M*_w_/*M*_n_^5^
1	600/1/0.048	45	Ag^0^	37	0.064	225	35.5	31.4	1.64
2	600/1/0.048	45	-	18	0.025	107	17.1	20.8	1.42
3	600/1/0.060	57	-	23	0.033	136	21.6	20.7	1.41

^1^ General conditions: T = 50 °C; *V*_tot_ = 15 mL; *t* = 9 h for entry 1 and 2 and T = 50 °C; *V*_tot_ = 20 mL; *t* = 9 h for entry 3. SI-ARGET ATRP with silver wire: *d* = 0.05 cm, *l* = 80 cm for entry 1; ^2^ Monomer conversion (Conv) and theoretical degree of polymerization (DP_n_,_theo_) calculated according to ^1^H NMR analysis of the samples withdrawn from the solution, DP_n_,_theo_ = (Conv · [DMAEMA]_0_)/[EBiB]_0_; ^3^ Apparent rate constant of propagation, calculated as a slope of the curve ln([M]_0_/[M]) = *f(t*) (illustrated in the Figure 2a); ^4^ *M*_n,theo_ = ([DMAEMA]_0_/[EBiB]_0_) Conv *M*_DMAEMA_ + *M*_EBiB_; ^5^ *M*_n,app_ and *M*_w_/*M*_n_ were determined by DMF GPC. Abbreviations: Ag^0^ SI-ARGET ATRP surface initiated activators regenerated by electron transfer atom transfer radical polymerization mediated with Ag^0^ as a reducing agent, DMAEMA—2-(dimethylamino)ethyl methacrylate, Cu^II^Br_2_/TPMA–copper(II) bromide/tris(2-pyridylmethyl)amine catalytic complex, EBiB—ethyl 2-bromoisobutyrate.

**Table 3:** Summary novel wood–polymer composites ^1^.

Entry	Monomer	[Cu^II^Br_2_]_0_, (ppm by wt)	Reducing Agent	Conv ^2^ (%)	*k*_p_^app 3^ (h^−1^)	DP_n_,_theo_^2^ (per chain)	*M*_n,theo_^4^ (×10^−3^)	*M*_n,app_^5^ (×10^−3^)	*M*_w_/*M*_n_^5^
1	MMA	72	Ag^0^	71	0.120	426	42.8	17.9	1.31
2 ^6^	DMAEMA	45	-	20	0.027	120	19.1	20.2	1.41

^1^ General conditions: T = 50 °C; *V*_tot_ = 15 mL; *t* = 9.5 h for entry 1, *t* = 9 h for entry 2; [Monomer]_0_/[EBiB]_0_/[Cu^II^Br_2_/TPMA]_0_ = 600/1/0.048. SI-ARGET ATRP with silver wire: *d* = 0.05 cm, *l* = 80 cm for entry; ^2^ Monomer conversion (Conv) and theoretical degree of polymerization (DP_n_,_theo_) calculated according to ^1^H NMR analysis of the samples withdrawn from the solution, DP_n_,_theo_ = (Conv · [Monomer]_0_)/[EBiB]_0_; ^3^ Apparent rate constant of propagation, calculated as a slope of the curve ln([M]_0_/[M]) = *f(t*) (illustrated in the Figure 3a,c); ^4^ *M*_n,theo_ = ([Monomer]_0_/[EBiB]_0_) Conv *M*_Monomer_ + *M*_EBiB_; ^5^ *M*_n,app_ and *M*_w_/*M*_n_ were determined by DMF GPC; ^6^ PMMA (entry 1) as an initiator for polymerization. Abbreviations: MMA—methyl methacrylate, DMAEMA—2-(dimethylamino)ethyl methacrylate EBiB—ethyl 2-bromoisobutyrate, TPMA—tris(2-pyridylmethyl)amine, Cu^II^Br_2_/TPMA—catalytic complex.

**Table 4:** Reduction ratios in the number of *S. aureus* and *E. coli* cells after 24 h incubation at 37 °C relative to viable bacteria on wood control.

Samples	Number of Disinfection Cycles	Survival Fraction	Log Reduction (CFU/mL)	% Reduction
Gram-positive *Staphylococcus aureus*				
Wood-PMMA-*b*-QPDMAEMA-Br	- *	0.008 ± 0.0005	1.9	99.17 ± 0.08
Wood-QPDMAEMA-Br	- *	0.00003 ± 0.0003	3.95	99.997 ± 0.13
	1	0.0046 ± 0.0005	2.32	99.54 ± 0.15
	2	0.027 ± 0.001	1.4	97.28 ± 0.83
Gram-negative *Escherichia coli*				
Wood-PMMA-*b*-QPDMAEMA-Br	- *	0.18 ± 0.02	0.73	81.88 ± 1.68
Wood-QPDMAEMA-Br	- *	0.07 ± 0.002	1.12	92.58 ± 0.54
	1	0.09 ± 0.001	1.04	90.97 ± 0.82
	2	0.23 ± 0.001	0.64	77.46 ± 0.54

* wood pretreated only with UV radiation (30 min).

**Table 5:** MIC and MBC of QPDMAEMA solution and antibiotics on *S. aureus* and *E. coli* strains.

Antibacterial Agent	*Escherichia coli*	*Staphylococcus aureus*
	MIC/MBC (µg/mL)	
QPDMAEMA	52.97/52.97	6.62/13.24
	**MIC/MBC** **(µg/mL)**	
Tetracycline	0.5/0.5	0.12/0.12
Gentamicin	1.9/1.9	0.9/0.9
Oxacillin	125/125	0.12/> 0.49
Rifampicin	3.91/3.91	< 0.01/> 0.06

## Data Availability

The data presented in this study are available on request from the corresponding author.

## Supplementary Materials

The following supporting information can be downloaded, Table S1. Estimation of the amount of available hydroxyl groups in the appropriate weight of ash wood depending on the cellulose content, Figure S1. GPC traces of PMMA, with different catalyst loadings [Cu^II^Br_2_]: (a) 273, (b) 182, (c) 90, (d) 72, (e) 9 ppm by wt. (Table 1), Figure S2. GPC traces of PDMAEMA received by ARGET ATRP with different catalyst loadings: (a) 45 ppm by wt. + Ag^0^ (b) 45 ppm by wt. and (c) 57 ppm by wt. (Table 2), Figure S3. GPC traces of PMMA-*b*-PDMAEMA (Table 3), Figure S4. ^1^H NMR spectrum of PMMA homopolymer (*M*_n_ = 17,900; *M*_w_/*M*_n_ = 1.31) after purification in CDCl_3_ (Table 1, entry 4), Figure S5. ^1^H NMR spectrum of PDMAEMA homopolymer (*M*_n_ = 20,800; *M*_w_/*M*_n_ = 1.42) after purification in CDCl_3_ (Table 2, entry 2), Figure S6. FT-IR spectra of native wood (untreated), and wood grafted with wood-PMMA-Br with different catalyst loadings, Figure S7. FT-IR spectra of native wood (untreated), and wood grafted with wood-PDMAEMA-Br with different catalyst loadings and after the quaternization reaction wood-QPDMAEMA-Br, Table S2. Fourier transform infrared spectroscopy (FTIR) analysis used to study chemical modification of ash wood Figure S8. SEM images composed of modified wood-PMMA-Br: (a) 273 ppm by wt, (b) 182 ppm by wt (c) 90 ppm by wt, (d) 72 ppm by wt and (e) 9 ppm by wt, viewed at different resolutions (100× 50× 20× 10×) focusing on the same spatial point. The photos show the presence of the polymer: it has formed complex structures around the conductive vessels of the wood, Figure S9. SEM images composed of modified wood-PDMAEMA-Br: (a) 45 ppm by wt + Ag^0^, (b) 45 ppm by wt and (c) 57 ppm by wt, viewed at different resolutions (100× 50× 20× 10×) focusing on the same spatial point. The photos show the presence of the polymer: it has formed complex structures around the conductive vessels of the wood, Figure S10. Characteristics of polymer materials brushes prepared by ATRP method with AFM topography images wood-PDMAEMA-Br: (a) 45 ppm by wt + Ag^0^, (b) 45 ppm by wt, and (c) 57 ppm by wt, Figure S11. Contact angle measurements for native wood and wood modified with polymers PMMA at 22 °C during 120 s (side chains synthesized by different concentrations of the catalytic complex), Figure S12. Contact angle measurements for native wood, wood modified with polymers at 22 °C during 15 s (side chains synthesized by different concentrations of the catalytic complex and using the DMAEMA monomer while presenting its reducing properties in the reaction system).

## Sample Availability

Samples of the compounds presented in the paper are available on request from the corresponding author.
