# Supplementary material for: A New Protocol for Ash Wood Modification: Synthesis of Hydrophobic and Antibacterial Brushes from the Wood Surface
Source: Molecules. 2022 Jan 28;27(3):890. doi: 10.3390/molecules27030890 (PMC8840146; doi:10.3390/molecules27030890)
Supplement: Supplementary file 1 [file molecules-27-00890-s001.zip › molecules-1527928-supplementary.pdf]

molecules

Supplementary Material

A New Protocol for Ash Wood Modification: Synthesis of Hydrophobic and Antibacterial Brushes from the Wood Surface

Angelika Macior[1], Izabela Zaborniak[2], Paweł Chmielarz[2,*], Joanna Smenda[3], Karol Wolski[3], Ewa Ciszkowicz[4] and Katarzyna Lecka-Szlachta[4]

[1] Doctoral School of Engineering and Technical Sciences at Rzeszow University of Technology, Al. Powstańców Warszawy 8, 35-959 Rzeszów, Poland; d519@stud.prz.edu.pl
[2] Department of Physical Chemistry, Faculty of Chemistry, Rzeszow University of Technology, Al. Powstańców Warszawy 6, 35-959 Rzeszów, Poland; i.zaborniak@prz.edu.pl; p_chmiel@prz.edu.pl
[3] Faculty of Chemistry, Jagiellonian University, Gronostajowa 2, 30-387 Kraków, Poland; joanna.smenda@doctoral.uj.edu.pl; wolski@chemia.uj.edu.pl
[4] Department of Biotechnology and Bioinformatics, Faculty of Chemistry, Rzeszow University of Technology, Al. Powstańców Warszawy 6, 35-959 Rzeszów, Poland; eciszkow@prz.edu.pl; szlachta@prz.edu.pl
* Correspondence: p_chmiel@prz.edu.pl; Tel.: +48-17-865-1809

Content

S1. Calculation of the amount of BriBBr initiator precursor to generate ATRP initiation sites on the wood surface.......3

S2. GPC analysis of PMMA, PDMAEMA and PMMA-*b*-PDMAEMA macromolecules..5

S3. Proton nuclear magnetic resonance (^{1}H NMR) spectroscopy of PMMA and PDMAEMA linear polymers.............8

S4. Fourier-transform infrared spectroscopy (FT-IR) analysis of ash wood specimens...10

S5. Scanning electron microscopy (SEM) analysis of ash wood specimens...13

S6. AFM analysis of PDMAEMA brushes grafted from silicon wafers 15

S7. Contact angle measurements..16

References..17

S1. Calculation of the amount of BriBBr initiator precursor to generate ATRP initiation sites on the wood surface

The key parameter is the modification of wood by esterification of the hydroxyl groups found on all major components wood cell wall (cellulose, hemicellulose and lignin). In fact, a significant part of –OH groups is unavailable or slightly reactive. This is the case of the –OH groups that are involved in hydrogen bonding, such as those found in regions of crystalline cellulose. Taking this into account, several assumptions are made. Wood mostly consists of 40 to 45% cellulose, 20 to 30% hemicellulose and 20 to 40% lignin.

For the purposes of calculating the amount of BriBBr use, several assumptions were made:

- The calculations assume that wood consists of 50% of cellulose (including the hydroxyl groups of other components), the remaining 50% of the weight of wood does not contain reactive hydroxyl groups.
- One unit of cellulose, the so-called anhydroglucose unit (AGU), has a molecular weight of 162.14 g / mol and contains three -OH groups (only one of these three hydroxyl groups can react).

Table S1. Estimation of the amount of available hydroxyl groups in the appropriate weight of ash wood depending on the cellulose content.

$[Cu^{II}Br_2]$, (ppm] by wt)	m_{wood} (g)	$m_{cellulose}$ (g)	$n_{cellulose}$ (mmol)	n_{OH} (mmol)	$n_{BriBBr} : n_{OH}$	n_{BriBB} (mmol)	m_{BriBB} (g)
preparation of a wood sample for MMA modification							
273	0.1980	0.0990	0.611	1.832	1	1.832	0.421
	0.1694	0.0847	0.520	1.157		1.157	0.360
182	0.1728	0.0864	0.533	1.599		1.599	0.367
	0.1822	0.0911	0.560	1.690		1.690	0.387
90	0.1661	0.0830	0.512	1.537		1.537	0.353
	0.1825	0.0912	0.169	1.688		1.688	0.388
72	0.1680	0.0840	0.518	1.554		1.554	0.357
	0.1752	0.0876	0.540	1.620		1.620	0.372
	0.1613	0.0806	0.499	1.499		1.499	0.343
	0.1621	0.0810	0.500	0.150		0.150	0.345
9	0.1689	0.0844	0.521	1.563		1.563	0.359
	0.1722	0.0861	0.530	1.590		1.590	0.366
preparation of a wood sample for DMAEMA modification							
45	0.2029	0.1014	0.626	1.877	1	1.877	0.431
	0.1992	0.0996	0.610	1.840		1.840	0.424
	0.2002	0.1001	0.600	1.900		1.900	0.426
45 + Ag^0	0.1909	0.0954	0.589	1.766		1.766	0.406
	0.1753	0.0876	0.540	1.620		1.620	0.373
	0.1820	0.0910	0.600	1.700		1.700	0.387
	0.1951	0.0975	0.601	1.805		1.805	0.415
	0.1539	0.0769	0.457	1.424		1.424	0.327
57	0.1840	0.0920	0.567	1.702		1.702	0.391
	0.1882	0.0941	0.580	1.740		1.740	0.400
	0.1653	0.0826	0.500	1.500		1.500	0.352
	0.1575	0.0787	0.485	1.457		1.457	0.335

S2. GPC analysis of PMMA, PDMAEMA and PMMA-*b*-PDMAEMA macromolecules

GPC analysis of PMMA macromolecules

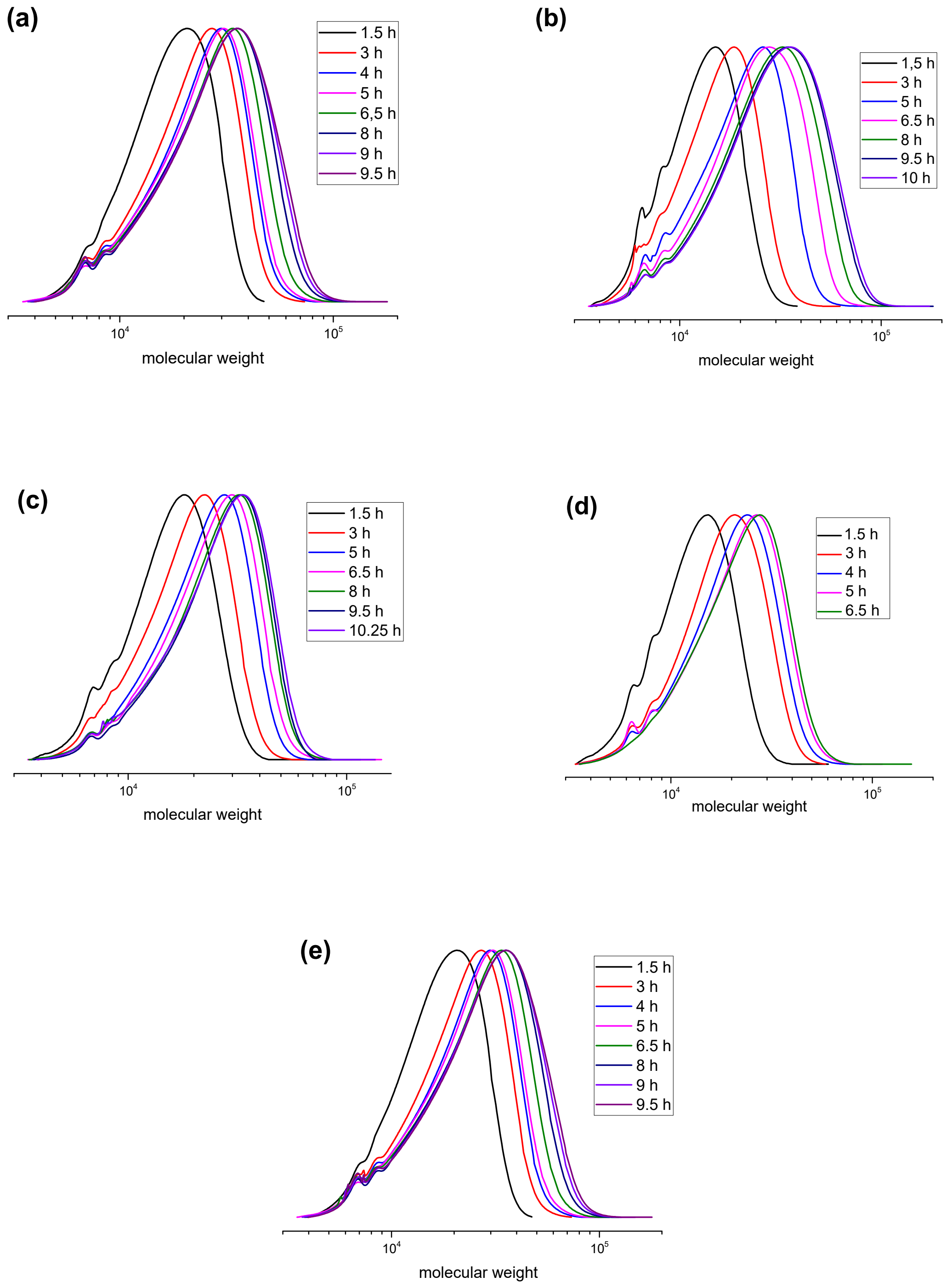

Figure S1. GPC traces of PMMA received by ARGET ATRP with different catalyst loadings: (a) 273, (b) 182, (c) 90, (d) 72, (e) 9 ppm by wt (**Table 1**).

GPC analysis of PDMAEMA macromolecules

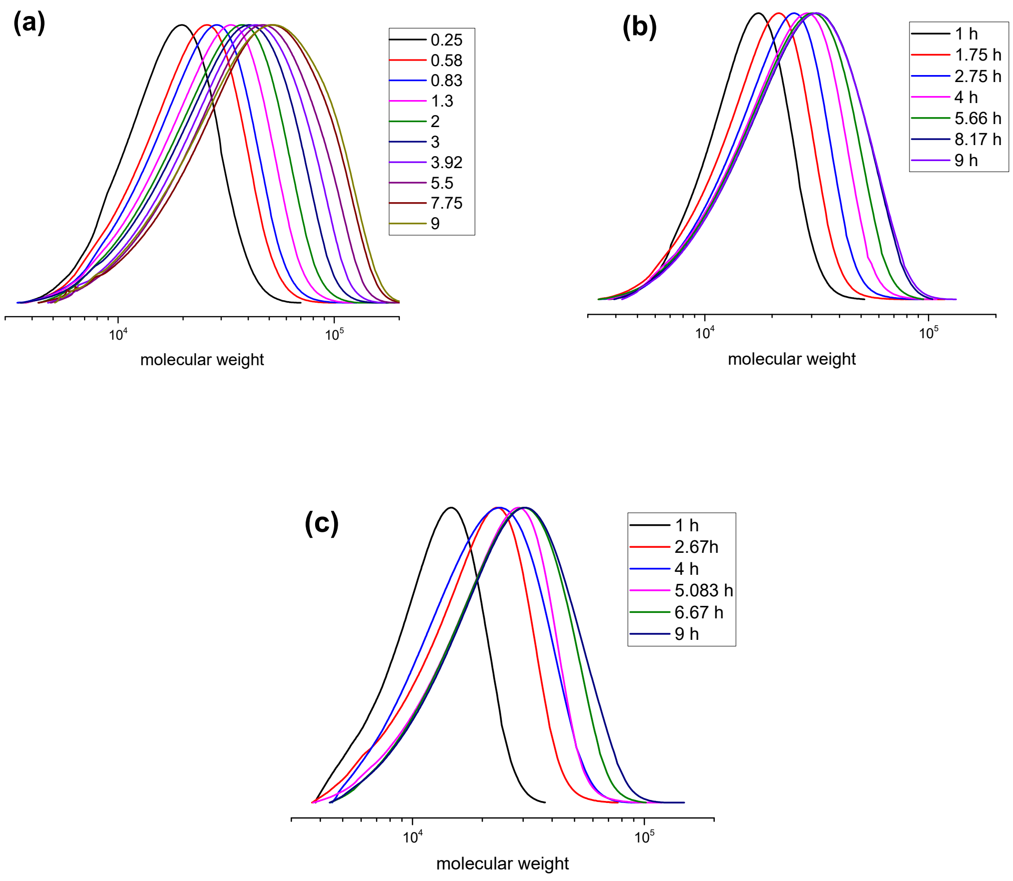

Figure S2. GPC traces of PDMAEMA received by ARGET ATRP with different catalyst loadings: (a) 45 ppm by wt + Ag^0, (b) 45 ppm by wt and (c) 57 ppm by wt (Table 2).

GPC analysis of PMMA-b-PDMAEMA macromolecules

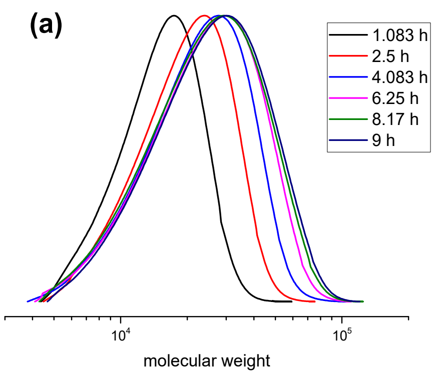

Figure S3. GPC traces of PMMA-*b*-PDMAEMA (**Table 3**).

S3. Proton nuclear magnetic resonance (¹H NMR) spectroscopy of PMMA and PDMAEMA linear polymers

The identified chemical shifts attributed to the characteristic groups of MMA units (**Figure S4)** indicate the formation of polymer chains, units which were assigned as follows: δ (ppm) = 0.69–1.17 (3H, CH_3–, α), 1.65–2.10 (2H, –CH_2–, β) and 3.45–3.75 (3H, CH_3–, a). The identified chemical shifts attributed to the characteristic groups of MMA units indicate the formation of polymer chains [1].

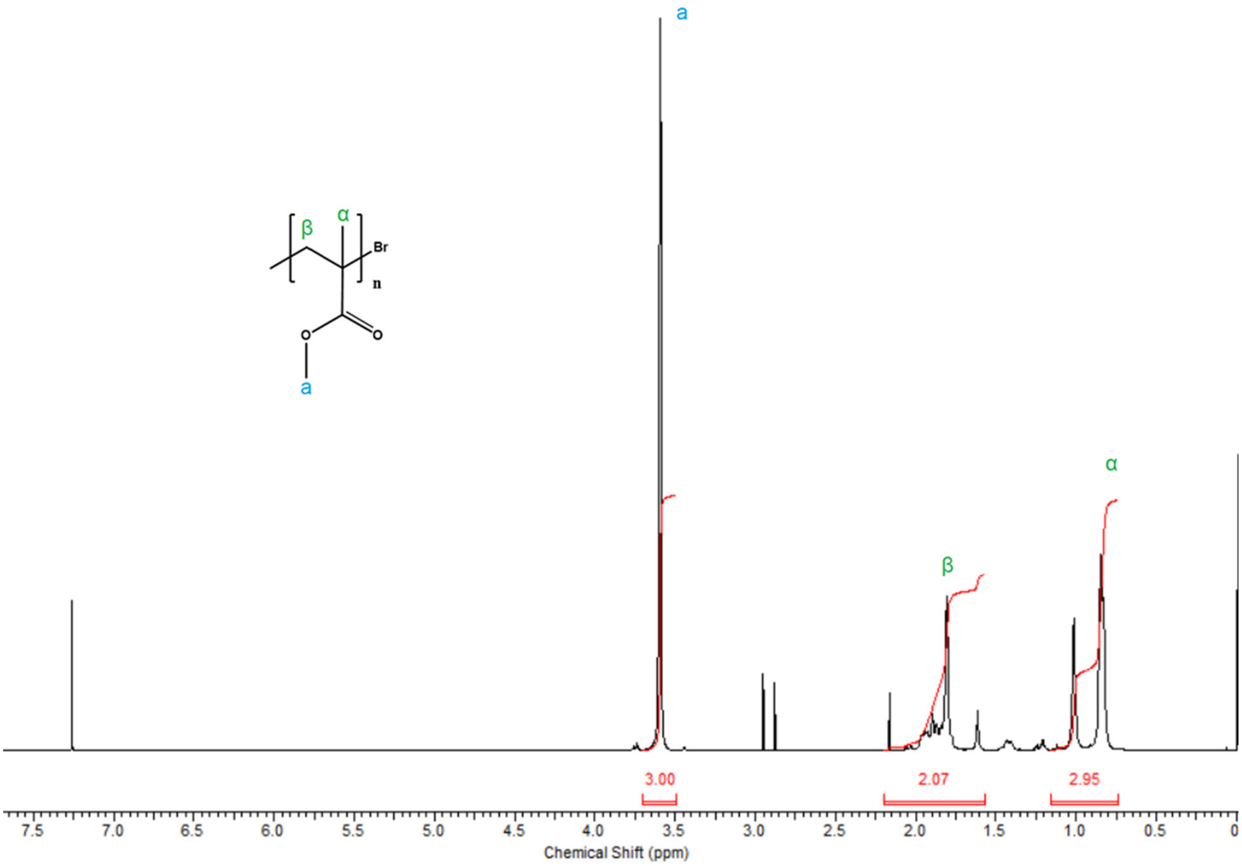

Figure S4. ¹H NMR spectrum of PMMA homopolymer (M_n = 17,900; M_w/M_n = 1.31) after purification in $CDCl_3$ (**Table 1, entry 4**).

The identified chemical shifts attributed to the characteristic groups of DMAEMA units **Figure S5** indicate the formation of polymer chains, units which were assigned as follows: δ (ppm) = 0.69–1.17 0.85–1.77 (3H, –CH_2CCH_3–, 3H, –CH_2 $(CH_2)_{10}CH_3$–, α), 1.75–2.04 (2H, –CH_2C–,β) and3.95–4.20 (2H, –OCH_2CH_2N–, a), 2.55–2.75 (2H, –OCH_2CH_2N–, b), 2.20–2.40 (6H, –$N(CH_3)_2$, c) . The identified chemical shifts attributed to the characteristic groups of DMAEMA units indicate the formation of polymer chains [2,3].

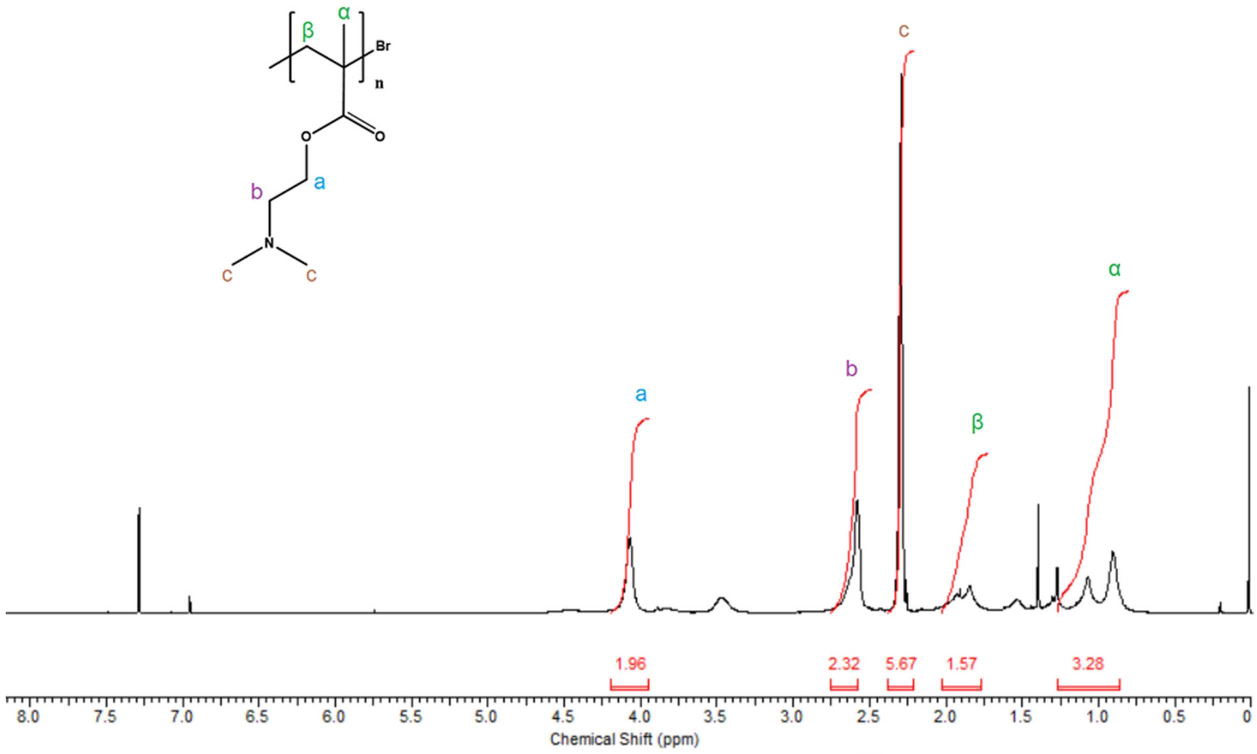

Figure S5.^{1}H NMR spectrum of PDMAEMA homopolymer (M_n = 20,800; M_w/M_n = 1.42) after purification in $CDCl_3$ (**Table 2, entry 2**).

S4. Fourier-transform infrared spectroscopy (FT-IR) analysis of ash wood specimens

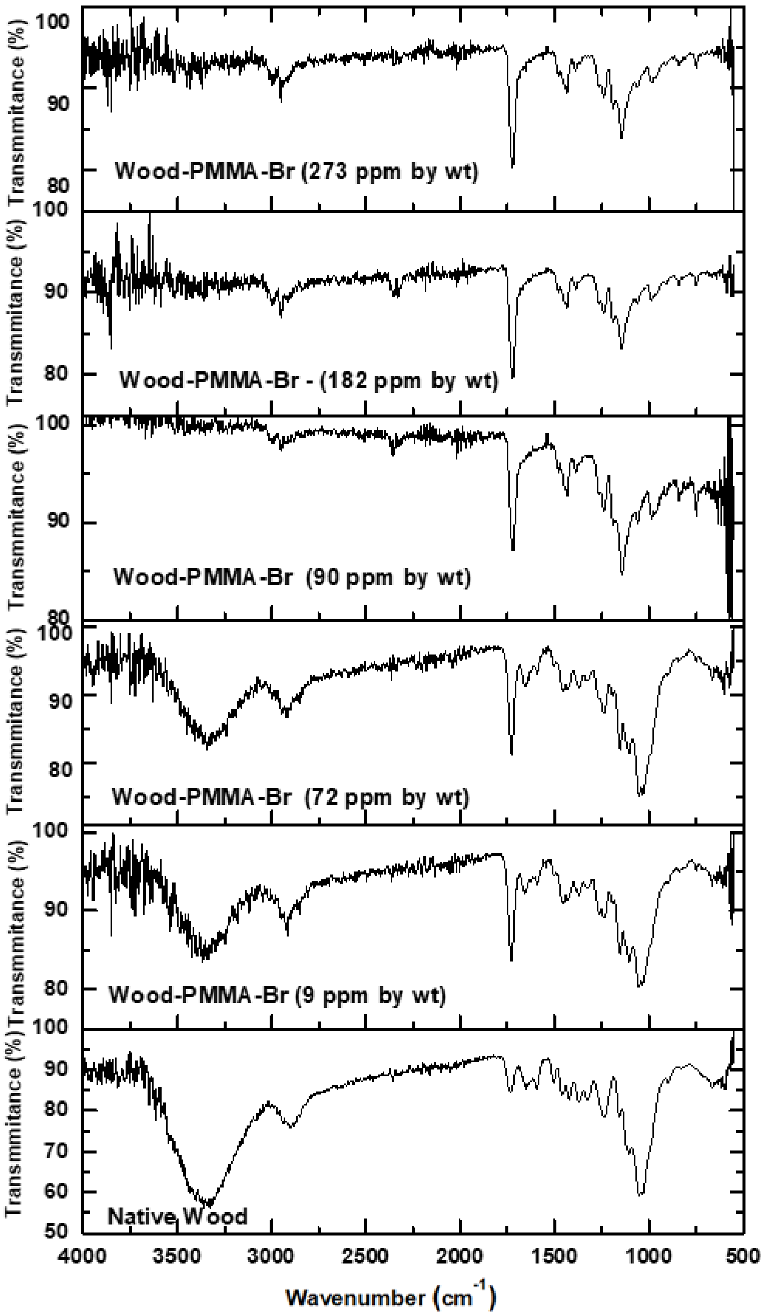

Figure S6. FT-IR spectra of native wood (untreated), and wood grafted with wood-PMMA-Br with different catalyst loadings.

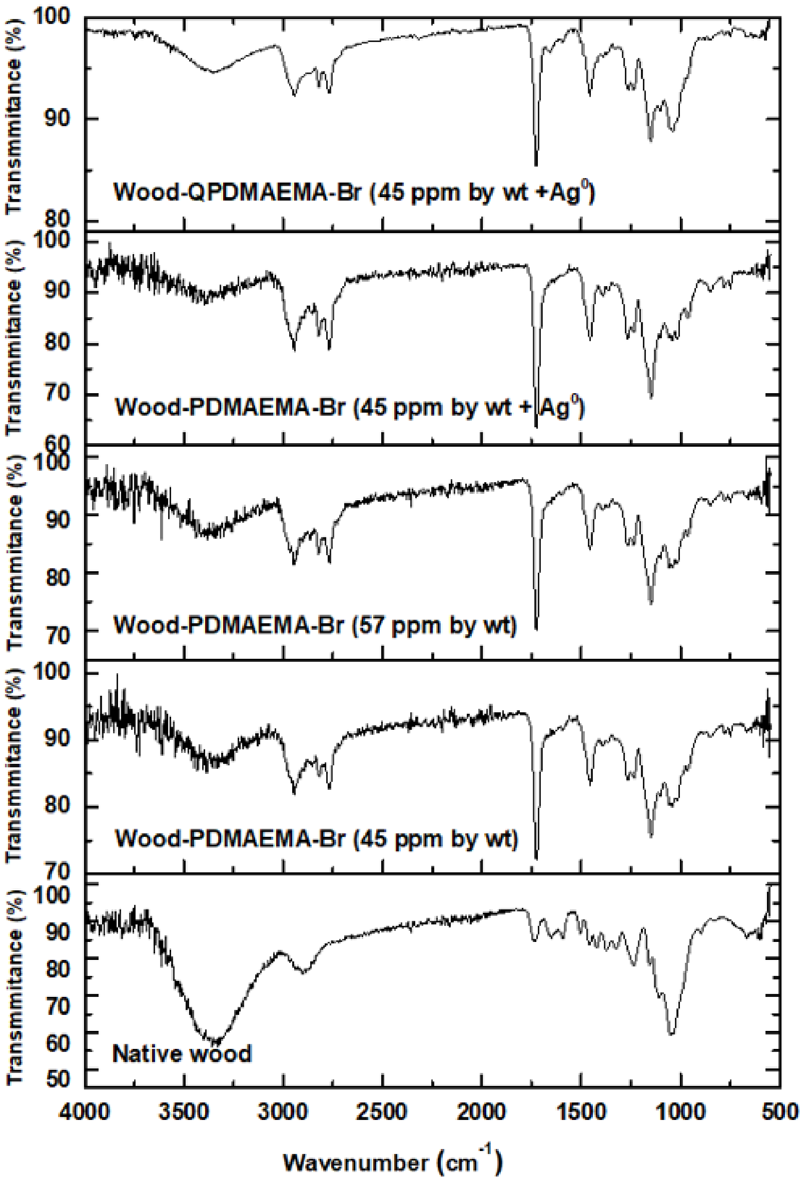

Figure S7. FT-IR spectra of native wood (untreated), and wood grafted with wood-PDMAEMA-Br with different catalyst loadings and after the quaternization reaction wood-QPDMAEMA-Br.

Table S2. Fourier transform infrared spectroscopy (FTIR) analysis used to study chemical modification of ash wood

Description of vibrations	Wavenumbers	References
In the FT-IR spectrum of Wood-Br		
stretching vibration of C=O group	1,741 cm^{-1}	[4,5]
stretching vibration of OH bond	3,400 cm^{-1}	
In the FT-IR spectrum of Wood-PMMA-Br		
C−H stretching of methyl groups	2,927–2,986 cm^{-1}	[6,7]
C=O stretching of the ester group	1,700–1,744 cm^{-1}	
CH_3 stretching	1,439 cm^{-1}	
$-OCH_3$ stretching	1,195 cm^{-1}	
In the FT-IR spectrum of Wood-PDMAEMA-Br		
C−H symmetric and asymmetric stretching of methyl and methylene groups	2,937 cm^{-1}	[5]
C−H stretching of $-N(CH_3)_2$ groups	2,825 and 2,767 cm^{-1}	
C−N stretching of $-N(CH_3)_2$ groups	1,151 cm^{-1}	
C=O stretching of the ester group	1,726 cm^{-1}	
In the FT-IR spectrum of Wood-QPDMAEMA-Br		
C–H stretching, $-CH_3$, $-CH_2-$ and $-N^+(CH_3)_2$	1,475, 2,929, 2,863 and 2,774 cm^{-1}	[8,9]

S5. Scanning electron microscopy (SEM) analysis of ash wood specimens

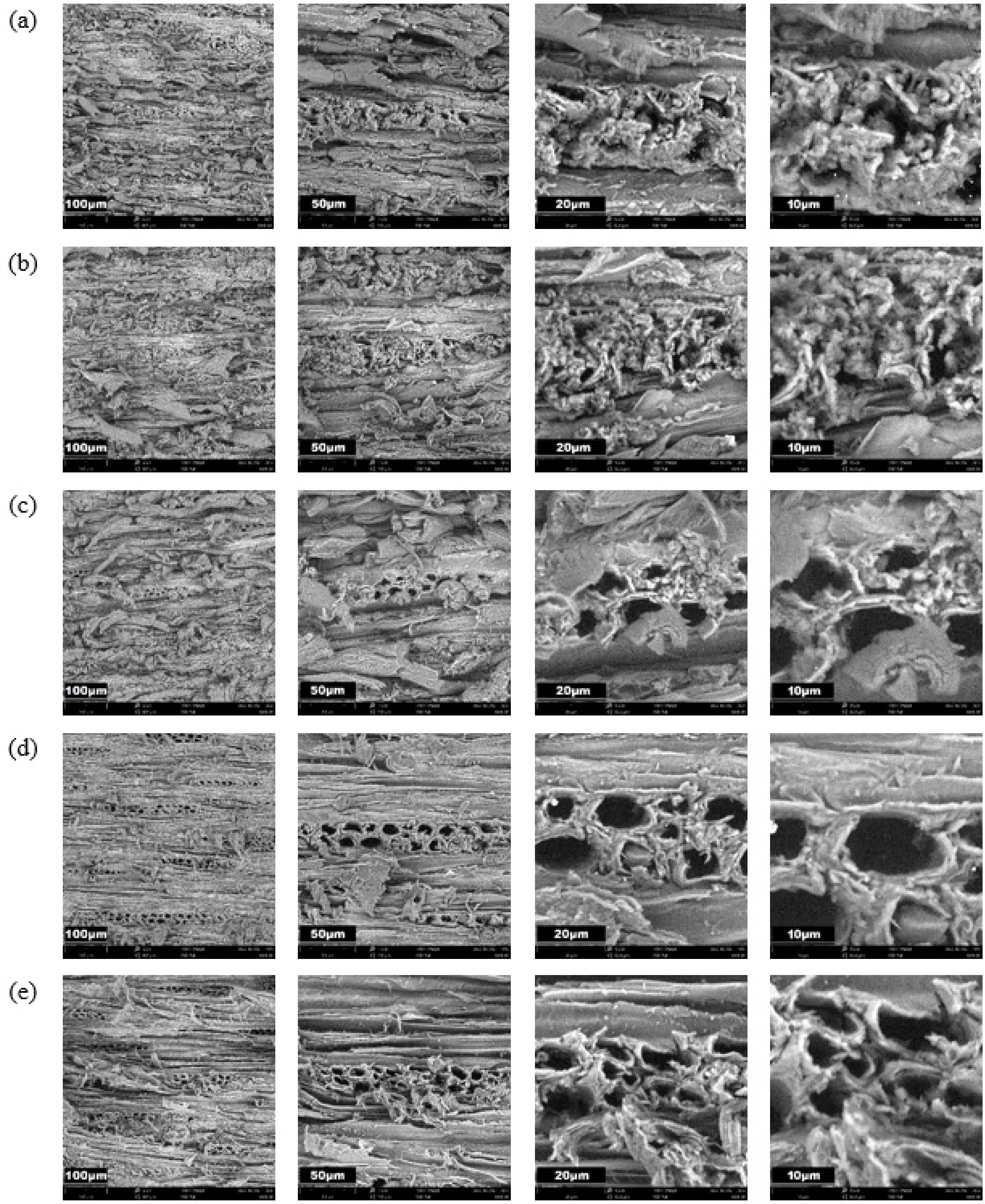

Figure S8. SEM images of modified wood-PMMA-Br: (a) 273 ppm by wt, (b) 182 ppm by wt (c) 90 ppm by wt, (d) 72 ppm by wt and (e) 9 ppm by wt, viewed at different magnifications (530× 1400× 4300× 7800×). The photos show the presence of the polymer: it has formed complex structures around vessels of the wood.

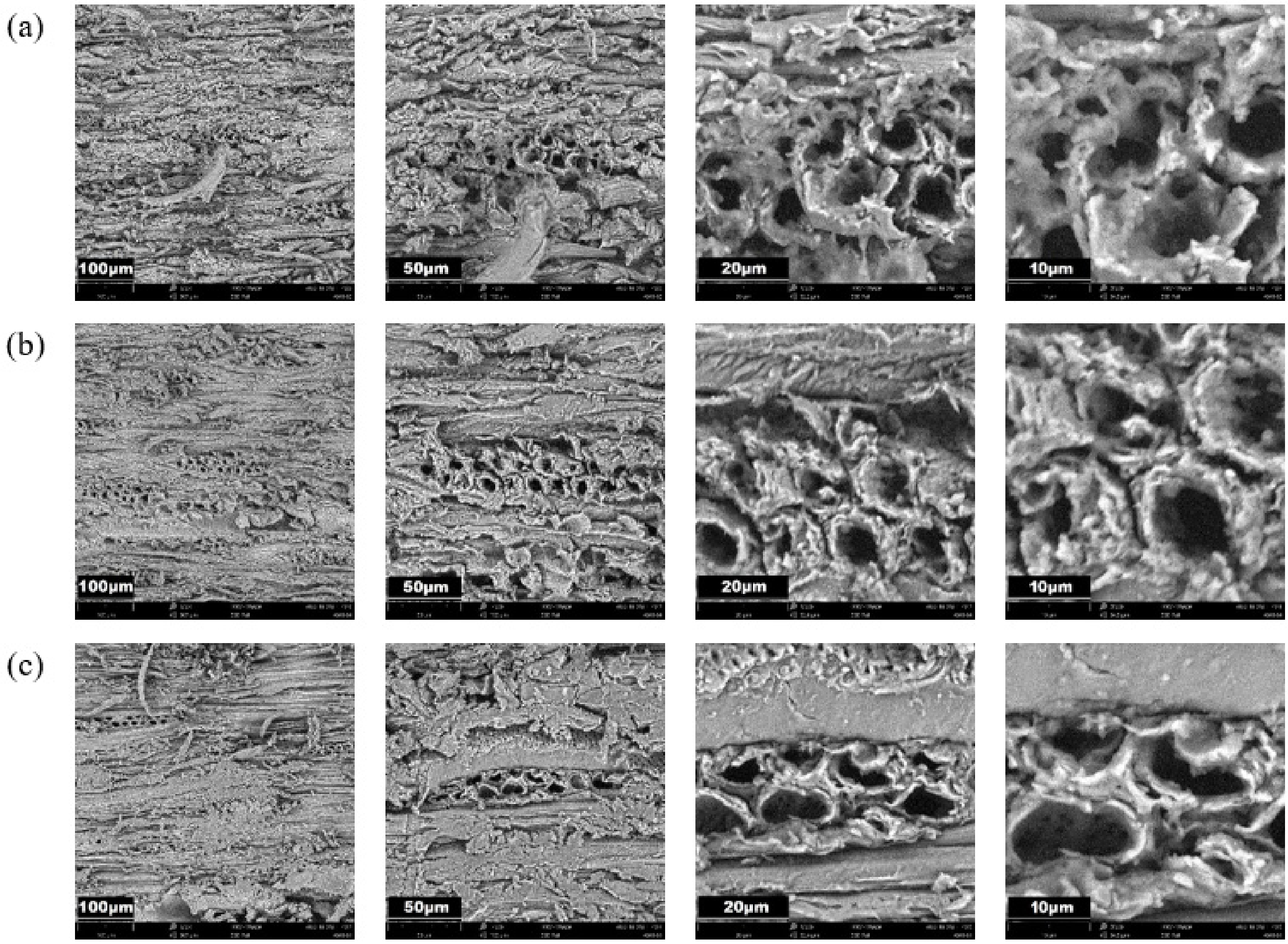

Figure S9. SEM images of modified wood-PDMAEMA-Br: (a) 45 ppm by wt + Ag^0, (b) 45 ppm by wt and (c) 57 ppm by wt, viewed at different magnifications (530× 1400× 4300× 7800×). The photos show the presence of the polymer: it has formed complex structures around vessels of the wood.

S6. AFM analysis of PDMAEMA brushes grafted from silicon wafers

To estimate the thickness of the polymer adhered to the wood surface, brominated silicon wafers were placed in the reaction mixture, assuming that the polymers grew evenly from both the silicon wafers and the wood initiation sites (**Table 2**).

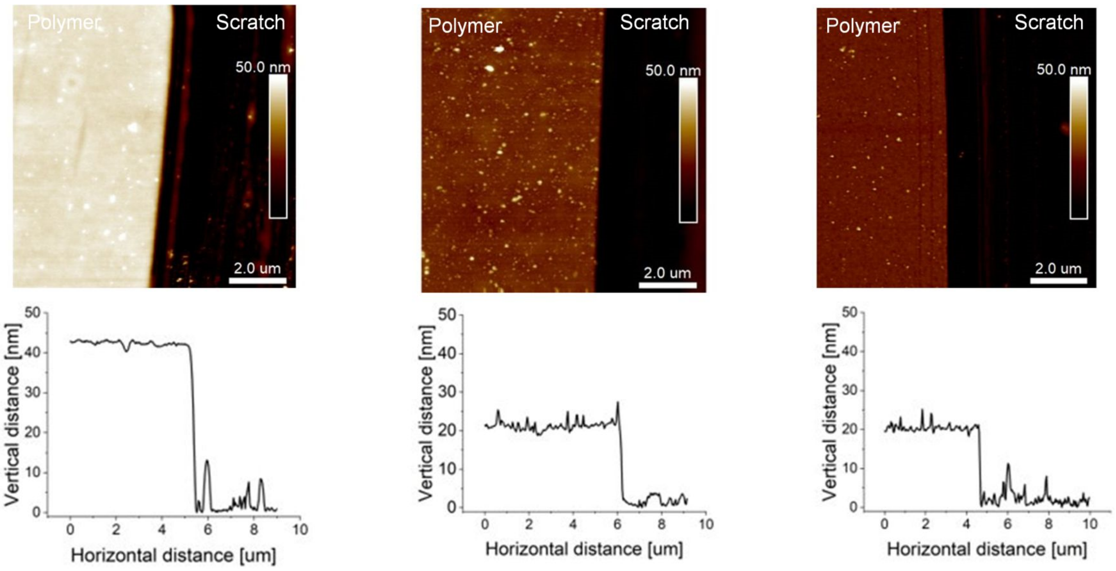

Figure S10. AFM images with representative cross-section profiles of polymer brushes obtained via SI-ATRP wood-PDMAEMA-Br: (a) 45 ppm by wt + Ag^0, (b) 45 ppm by wt, and (c) 57 ppm by wt.

S7. Contact angle measurements

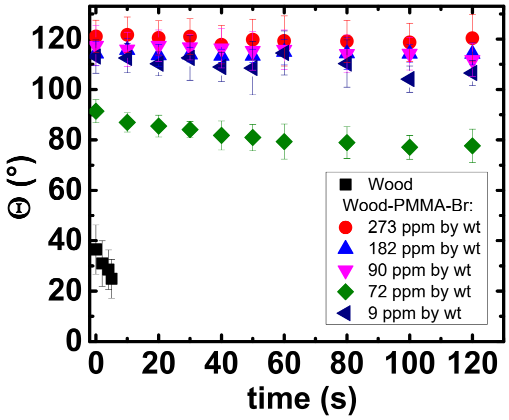

Figure S11. Contact angle measurements for native wood and wood modified with PMMA at 22°C during 120 s (side chains synthesized by different concentrations of the catalytic complex).

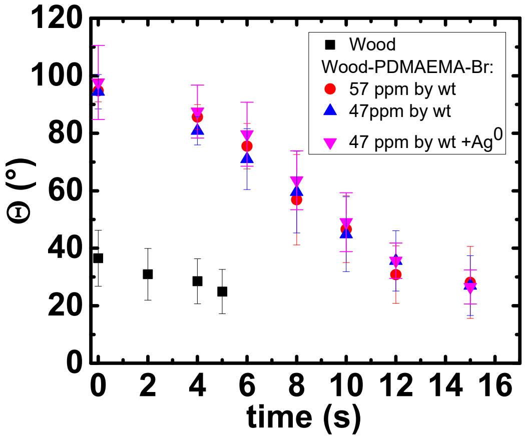

Figure S12. Contact angle measurements for native wood, wood modified with polymers at 22°C during 15 s (side chains synthesized by different concentrations of the catalytic complex and using the DMAEMA monomer while presenting its reducing properties in the reaction system).

References

1. Brar, A.S.; Singh, G.; Shankar, R. Structural investigations of poly(methyl methacrylate) by two-dimensional NMR. *J. Mol. Struct.* **2004**, *703*, 69-81.
2. Chrysostomou, V.; Pispas, S. Stimuli-responsive amphiphilic PDMAEMA-*b*-PLMA copolymers and their cationic and zwitterionic analogs. *J. Polym. Sci. A: Polym. Chem* **2018**, *56*, 598-610.
3. Dong, Z.; Wei, H.; Mao, J.; Wang, D.; Yang, M.; Bo, S.; Ji, X. Synthesis and responsive behavior of poly(*N*,*N*-dimethylaminoethyl methacrylate) brushes grafted on silica nanoparticles and their quaternized derivatives. *Polymer* **2012**, *53*, 2074-2084.
4. Sui, X.; Yuan, J.; Zhou, M.; Zhang, J.; Yang, H.; Yuan, W.; Wei, Y.; Pan, C. Synthesis of cellulose-*graft*-poly(*N*,*N*-dimethylamino-2-ethyl methacrylate) copolymers via homogeneous ATRP and their aggregates in aqueous media. *Biomacromolecules* **2008**, *9*, 2615-2620.
5. Yang, Y.; Wang, J.; Zhang, J.; Liu, J.; Yang, X.; Zhao, H. Exfoliated graphite oxide decorated by PDMAEMA chains and polymer particles. *Langmuir* **2009**, *25*, 11808-11814.
6. Ramesh, S.; Leen, K.H.; Kumutha, K.; Arof, A.K. FTIR studies of PVC/PMMA blend based polymer electrolytes. *Spectrochim. Acta A Mol. Biomol. Spectrosc.* **2007**, *66*, 1237-1242.
7. Rajendran, S.; Uma, T. Lithium ion conduction in PVC–$LiBF_4$ electrolytes gelled with PMMA. *J. Power Sources* **2000**, *88*, 282-285.
8. Tu, Q.; Tian, C.; Ma, T.; Pang, L.; Wang, J. Click synthesis of quaternized poly(dimethylaminoethyl methacrylate) functionalized graphene oxide with improved antibacterial and antifouling ability. *Colloids Surf. B: Biointerfaces* **2016**, *141*, 196-205.
9. Yao, D.; Guo, Y.; Chen, S.; Tang, J.; Chen, Y. Shaped core/shell polymer nanoobjects with high antibacterial activities via block copolymer microphase separation. *Polymer* **2013**, *54*, 3485-3491.
